# FABP6 Expression Correlates with Immune Infiltration and Immunogenicity in Colorectal Cancer Cells

**DOI:** 10.1155/2022/3129765

**Published:** 2022-08-17

**Authors:** Wenping Lian, Zhongquan Wang, Yajie Ma, Yalin Tong, Xinyu Zhang, Huifang Jin, Shuai Zhao, Ruijing Yu, Shaotan Ju, Xinyun Zhang, Xiaona Guo, Tao Huang, Xianfei Ding, Mengle Peng

**Affiliations:** ^1^Department of Clinical Laboratory, Henan Provincial Third People's Hospital, Zhengzhou, 450006 Henan, China; ^2^Department of Neurology, Henan Provincial Third People's Hospital, Zhengzhou, 450006 Henan, China; ^3^Department of Digestion, The First Affiliated Hospital of Zhengzhou University, Zhengzhou, 450052 Henan, China; ^4^Department of Medical Affair, Henan Provincial Third People's Hospital, Zhengzhou, 450006 Henan, China; ^5^Department of Blood Transfusion, The First Affiliated Hospital of Zhengzhou University, Zhengzhou, 450052 Henan, China; ^6^Department of Anorectal Surgery, Henan Provincial Third People's Hospital, Zhengzhou, 450006 Henan, China; ^7^Medical School, Huanghe Science and Technology University, 666 Zi Jing Shan Road, Zhengzhou, 450000 Henan, China; ^8^General ICU, The First Affiliated Hospital of Zhengzhou University, Zhengzhou 450052, China

## Abstract

**Background:**

Immune checkpoint inhibitors (ICIs) have rapidly revolutionized colorectal cancer (CRC) treatment, but resistance caused by the heterogeneous tumor microenvironment (TME) still presents a challenge. Therefore, it is necessary to characterize TME immune infiltration and explore new targets to improve immunotherapy.

**Methods:**

The compositions of 64 types of infiltrating immune cells and their relationships with CRC patient clinical characteristics were assessed. Differentially expressed genes (DEGs) between “hot” and “cold” tumors were used for functional analysis. A prediction model was constructed to explore the survival of CRC patients treated with and without immunotherapy. Finally, fatty acid-binding protein (FABP6) was selected for in vitro experiments, which revealed its roles in the proliferation, apoptosis, migration, and immunogenicity of CRC tissues and cell lines.

**Results:**

The infiltration levels of several immune cells were associated with CRC tumor stage and prognosis. Different cell types showed the synergistic or antagonism infiltration patterns. Enrichment analysis of DEGs revealed that immune-related signaling was significantly activated in hot tumors, while metabolic process pathways were altered in cold tumors. In addition, the constructed model effectively predicted the survival of CRC patients treated with and without immunotherapy. FABP6 knockdown did not significantly alter tumor cell proliferation, apoptosis, and migration. FABP6 was negatively correlated with immune infiltration, and knockdown of FABP6 increased major histocompatibility complex (MHC) class 1 expression and promoted immune-related chemokine secretion, indicating the immunogenicity enhancement of tumor cells. Finally, knockdown of FABP6 could promote the recruitment of CD8+ T cells.

**Conclusion:**

Collectively, we described the landscape of immune infiltration in CRC and identified FABP6 as a potential immunotherapeutic target for treatment.

## 1. Introduction

Colorectal cancer (CRC) is becoming one of the most common types of cancer worldwide, and its incidence is lower than only lung and liver cancer. In recent years, the morbidity and mortality rates of CRC have increased significantly. Early-stage CRC patients can be cured by surgical resection [1]. Due to the metastasis and recurrence of CRC tumors, the prognosis of patients with advanced CRC is poor despite the use of chemotherapy, surgical resection, radiotherapy, or immunotherapy [2]. Therefore, it is important to investigate the pathogenesis of CRC and identify new therapeutic strategies.

In recent studies, the tumor microenvironment (TME) has attracted increasing attention as a target for tumor therapy. Tumor cells in the TME can influence the development and progression of cancer by interacting with surrounding cells through the circulatory and lymphatic systems [3]. Except for malignant cells, immune cells, extracellular matrix (ECM), blood vessels, adipocytes, fibroblasts, lymphocytes, and signaling molecules are present in the TME [4]. These cells can exert antitumor or protumor activities in the TME. Immune cells and tumor cells were in the spotlight in previous studies, which greatly advanced this field. Also, many studies have found that other components in the TME play crucial roles in tumor progression. For instance, the ECM, which consists of a large number of glycoproteins, collagens, enzymes, and other macromolecules, can influence cell adhesion, proliferation, and communication [5]. Adipocytes, as a major component of the TME, can provide fatty acids for tumor growth and promote the homing, migration, and invasion of ovarian cancer cells [6]. Cancer-associated fibroblasts (CAFs) represent a heterogeneous group of stromal cells in the TME and are vital sources of growth factors and cytokines that promote tumor progression and migration, stimulating epithelial-mesenchymal transition (EMT), and immunosuppression [7–9].

Studies have shown that the TME represents a complex and dynamic milieu of both cellular and acellular components with synergistic responses and functions in cancer progression [10]. Tumor phenotypes are determined not only by the neoplastic cell component but also by the immunologic milieu within the TME, which facilitates the evasion of the host immune response and suppress effector T cell function [11]. The emergence of next-generation sequencing (NGS) technologies and the development of bioinformatics tools have enabled the characterization of multidimensional maps of genomic changes in common cancers. Immunogenomic analysis can provide a comprehensive view of the cellular composition of immune infiltrates in the TME [12, 13]. The immune infiltration pattern determines the immunophenotype, tumor escape mechanisms, and antitumor or protumor activity of the TME in tumor development and progression [14]. Therefore, it is critical to explore the cell composition and molecular mechanisms of the TME which affect tumor progression and immune response. Analysis of the levels of different tumor-infiltrating immune cell types in patients may be a new strategy for identifying new biomarkers and therapeutic targets [15–17].

In this study, we revealed the landscape of 64 cell types in CRC. We evaluated the clinical significance of these cells and explored the immune infiltration pattern. We found that some infiltrating cells were significantly related to tumor-node-metastasis (TNM) stage and clinical stage, as well as the overall survival (OS) and progression-free survival (PFS) of CRC patients. The constructed model predicted the OS in patients receiving immunotherapy treatment. In addition, our results revealed that knockdown of FABP6 increased the immunogenicity of tumor cells and promoted the secretion of immune-related chemokines, resulting in the recruitment of CD8+ T cells. Collectively, our study provides a new insight into the immune infiltration of CRC and identifies FABP6 as a potential therapeutic target for immunotherapy treatment.

## 2. Materials and Methods

### 2.1. Ethic Statement

Thirty CRC specimens were obtained from Henan Provincial Third People's Hospital and the First Affiliated Hospital of Zhengzhou University after surgical treatment and stored at -80°C for further use. All participants signed the informed consent form approved by the ethics committee of Henan Provincial Third People's Hospital (Ethics No. 2019-szsyky-02) and the First Affiliated Hospital of Zhengzhou University (Ethics No. 2021-KY-0147-002).

### 2.2. Data Collection and Estimation of Stromal and Immune Scores

The gene expression data of CRC samples and the corresponding clinical information of the patients were downloaded from UCSC XENA (https://xenabrowser.net/datapages/?dataset=TCGA.COADREAD.sampleMap%2FHiSeqV2&host=https%3A%2F%2Ftcga.xenahubs.net&removeHub=https%3A%2F%2Fxena.treehouse.gi.ucsc.edu%3A443) to estimate immune infiltration. The data type was log_2_(*x* + 1) RSEM normalized count. Clinicopathological data for the corresponding patients, including sex, race, age, tumor location, histology classification, differentiation grade, tumor stage, and survival information, were also retrieved from the database. The ESTIMATE algorithm was used to normalize expression matrix for calculating the stromal and immune scores [18]. For validation of gene signatures in predicting the response of immunotherapy, we downloaded the datasets of patients with metastatic urothelial cancer treated with atezolizumab (anti-PD-L1mAb) through the available link provided in the paper (http://research-pub.gene.com/IMvigor210CoreB iologies/) and patients with melanomas treated with pembrolizumab or nivolumab in Gene Expression Omnibus (GEO) database with accession number GSE78220.

### 2.3. Evaluation and Correlation of Immune Infiltration and Clinical Parameters

Tumor-infiltrating cells were calculated using the xCell algorithm [19]. OS and PFS were used as the primary prognostic endpoints and were estimated by the Kaplan-Meier survival method. Based on the level of infiltration of each cell type, patients were classified into two groups, and prognoses for each group were examined. Then, a method (maximally selected rank statistics) was performed to select the best score cutoff for grouping patients by using the R package “maxstat.” The log-rank tests were used to compare the survival outcomes between groups.

### 2.4. Consensus Clustering

Consensus clustering of 64 immune cell types was performed with the R package “ConsensusClusterPlus” with reps = 100, pfeature = 1, and pItem = 0.8. The optimal number of clusters was determined according to the heatmap and delta diagram.

### 2.5. Identification of Differentially Expressed Genes (DEGs)

DEGs between “hot” tumor group vs. “cold” tumor group were identified by using the R package “edgR,” with the criteria of false discovery rate (FDR) and adjusted *P* value < 0.05 and absolute value of log_2_ (fold change) > 1. And a heatmap and volcano plot were used to visualize the expression patterns of DEGs through the R packages “heatmap” and “ggplot2.”

### 2.6. Gene Ontology (GO) and Kyoto Encyclopedia of Genes and Genomes (KEGG) Enrichment Analysis of DEGs

Functional enrichment analysis of DEGs was performed with the R package “clusterProfiler”; the identified GO terms represented the enriched biological processes (BPs), molecular functions (MFs), and cellular components (CCs). We also performed KEGG enrichment analysis. A *P* value or *q* value less than 0.05 was used as the cutoff, and the top 10 GO terms and 30 KEGG terms were visualized with the R package “ggplot2.”

### 2.7. Construction of the Protein-Protein Interaction (PPI) Network

The PPI network was retrieved from the Search Tool for the Retrieval of Interacting Genes/Proteins (STRING) database and reconstructed via Cytoscape software. The connectivity degree of each node with 10 or more nodes was chosen for the following analysis. Then, clusters that based on topology to locate densely connected regions were identified by Molecular COmplex DEtection (MCODE).

### 2.8. Construction of Prediction Model

To construct the prediction model, DEGs were used, and CRC samples from The Cancer Genome Atlas (TCGA) were divided into two groups with the same mortality rate with the R package “caret.” Then, univariate Cox regression analysis was utilized to select survival-related genes by applying the R package “survival.” After that, the least absolute shrinkage and selection operator (LASSO) regression analysis was used to select the best combination of the gene sets by applying the R package “glm.” Multivariate Cox regression analysis was used to optimize the model, and genes with *P* < 0.05 were selected to construct the model. For the validation cohort, we excluded three genes (NCBP2, DECR1, and NUDT13) because of low expression, and the remaining genes were used to construct the model.

### 2.9. Cell Culture

CRC cell lines (HCT116 and SW1116) were purchased from the Chinese Academy of Sciences Cell Repertoire (Shanghai, China). Cells were cultured with Roswell Park Memorial Institute- (RPMI-) 1640 medium containing 10% fetal bovine serum, 100 units/mL penicillin, and 100 *μ*g/mL streptomycin (Thermo Fisher Scientific, Waltham, MA, USA) at 37°C with 5% CO_2_ in the humidified incubator.

### 2.10. Small Interfering RNA (siRNA) Transfection

HCT116 and SW1116 cells were cultured in RPMI-1640 medium containing 10% FBS and 1% penicillin and streptomycin in a constant temperature incubator at 37°C with 5% CO_2_. The cells were seeded in 12-well plates at a concentration of 1.5 × 10^5^ and transfected with siRNA the next day. The medium of each well was replaced with 800 *μ*L serum-free medium, and then, a 200 *μ*L mixture of Opti-MEM+2.5 *μ*L Lipofectamine 2000+5 *μ*L negative control (NC) or si-FABP6 was added to each well. The plate was gently shaken and placed in the incubator. The medium was replaced with 1 mL of complete medium containing serum and antibiotics after 8 h transfection, and the cells were collected at 48 h for subsequent experiments. The sequences of the human FABP6 siRNAs were as follows: si1-FABP6: 5′-GCCCGCAACUUCAAGAUCGTTCGAUCUUGAAGUUGCGGGCTT-3′ and si2-FABP6: 5′-GGAGAGUGAGAAGAAUUAUTTAUAAUUCUUCUCACUCUCCTT-3′ (Sangon Biotech, Shanghai, China).

### 2.11. Quantitative Real-Time PCR (qRT-PCR)

Total cellular RNA was extracted using TRIzol reagent (Takara, Japan). The concentration and purity of total RNA were determined, and then, 1 *μ*g of total RNA was reverse transcribed into cDNA with Prime Script RT Master Mix (Takara). The primers used in this study are listed in Supplementary Table 1. qRT-PCR analysis was carried out using the SYBR Premix Ex Taq™ kit (Takara). GAPDH was used as the internal control.

### 2.12. Western Blot

Total protein of cell lines was extracted with RIPA buffer (Beyotime, Beijing, China) and quantified by BCA Protein Assay Kit (Beyotime). Then, 30 *μ*g protein samples were dissolved using SDS-PAGE electrophoresis and transferred on PVDF membranes (ABclonal, Wuhan, China). After blocking with 5% skimmed milk dissolved in TBST for 2 hours, the membranes were incubated overnight at 4°C with the primary antibody (anti-FABP6, 1 : 1000 dilution (Proteintech, 13781-1-AP, Wuhan, China)). Following that, a secondary antibody was labeled by horseradish peroxidase (HRP) (ABclonal, Wuhan, China) for 2 h at room temperature, and Tubulin was used as internal control. Finally, the proteins were detected with an ECL kit (Beyotime).

### 2.13. Cell Counting Kit 8 (CCK-8) Analysis

For the CCK-8 assay, 4 × 10^3^ tumor cells were seeded in each well of a 96-well plate. After 24 h, 48 h, and 72 h, the supernatant of tumor cells was discarded and replaced with 100 *μ*L fresh complete medium containing 10 *μ*L diluted CCK-8 reagent. The absorbance at 450 nm was detected with a microplate reader.

### 2.14. Flow Cytometry

Cells were harvested through trypsinization and washed twice with cold PBS. Then, cells were centrifuged at 1500 rpm for 5 min, the supernatant was discarded, and the pellet was resuspended in 1× binding buffer at a density of 1 × 10^6^ cells per mL. Subsequently, 5 *μ*L of Annexin V-fluorescein isothiocyanate (FITC) and 5 *μ*L of propidium iodide (PI) (#640914, BioLegend, San Diego, CA, USA) were added. To detect HLA-A/B/C expression, tumor cells were labeled with 5 *μ*L HLA-A/B/C-APC (#311409) and the sample was incubated in the dark at room temperature for 15 min. Cell apoptosis and HLA-A/B/C expression were measured using a flow cytometer (BD, Accuri, C6).

### 2.15. Wound Scratch Assay

A total of 2 × 10^5^ cells/well (three replicates per group) were plated in a 6-well plate and grown to confluence. The media was removed, and the cells were rinsed with PBS. The cell monolayers were scratched using a sterile 1 mL pipette tip and washed twice with serum-free medium to remove the floating cells. Then, the cells were cultured in complete medium with 10% FBS. Images were taken after 0 h, 24 h, 48 h, and 72 h by using a microscope. The migration ability of the cells was calculated according to the healed area.

### 2.16. Transwell-Based Assays

For the migration assay, transwell assay was performed using transwell chambers (8 *μ*m pore; Corning, NY, USA). For the invasion assay, transwell chambers were precoated with Matrigel (Corning, NY, USA). The HCT116 and SW1116 cells (2 × 10^4^ for migration assay, 1 × 10^5^ for invasion analysis) in serum-free medium were seeded into the upper chamber, whereas the lower chamber was supplemented with medium with 10% fetal bovine serum. After incubating for 48 hours, migrated or invasive cells were fixed with 4% paraformaldehyde, stained with crystal violet for 30 min, and counted under five independent visual fields.

For the migration of T cells, CD8+ T cells were isolated from the peripheral blood of healthy donors and stimulated with CD3 and CD28 beads (1 *μ*g/mL, Sigma, Santa Clara, CA, USA) for 3 days. Then, a total of 8 × 10^4^ CD8+ T cells in 300 *μ*L serum-free medium were seeded into the upper chamber of the insert, and 700 *μ*L of tumor supernatant derived from HCT116 or SW1116 cells was added to the lower chamber. Following 4 h of incubation at 37°C, CD8+ T cells in the lower chamber were counted with an inverted microscope.

### 2.17. Immunohistochemistry (IHC) Assays

For the IHC analysis, the paraffin-embedded tissue was sectioned at a thickness of 3 *μ*m and placed on poly-L-lysine-coated slides. The slides were dried overnight at 60°C. The sections were deparaffinized via two changes of xylene. After blocking endogenous peroxidase activity with 3% hydrogen peroxide in methanol, antigen retrieval was performed by heating the slides in 10 mmol/l citrate buffer (pH 6) using a water bath. The sections were then incubated with the primary antibody (anti-CD3, 1 : 200 dilution (Abcam, ab1669, Cambridge, MA, USA) and anti-FABP6, 1 : 500 dilution (Proteintech, 13781-1-AP, Wuhan, China)) at 37°C for 30 min. Thereafter, the sections were rinsed with Tris-buffered saline (TBS) three times, incubated with the secondary antibody at room temperature for 30 min, and washed with TBS. Finally, 3,3′-diaminobenzidine (DAB) was used to illuminate the positive staining signals, and hematoxylin was used for counterstain. The positive staining signals were classified into 4 grades according to standard procedures.

### 2.18. Immunofluorescence

Cells (1 × 10^4^) were washed with PBS three times and fixed with 4% paraformaldehyde for 20 min. Then, Triton X-100 (1%) was added to permeabilize the cells for 10 min, and goat serum was used for blocking for 30 min. After that, cells were stained with primary antibodies: anti-HLA-A/B/C (1 : 500) (ab225636, Abcam, Cambridge, MA, USA) overnight at 4°C. After washing with PBS three times, the cells were incubated with fluorescence conjugated secondary antibodies. Next, the cells were stained with DAPI for 10 min, and images were observed under a fluorescence microscope.

### 2.19. ELISA

Tumor cells (2 × 10^5^) were seeded in 6-well plates after 48 h, and the supernatant was harvested. ELISA kits (CCL5, #70-EK1129-24, CXCL9, #70-EK1143-24) were purchased from MultiSciences. First, 200 *μ*L of 1× assay diluent was added to the wells of a 96-well plate and incubated for 1 h. Then, the supernatants or standard solutions were added according to the manufacturer's instructions. After incubation, the supernatant was removed, and the cells were washed with PBS 3 times. Then, 100 *μ*L of 1× detection antibody was added, followed by 100 *μ*L of 1× avidin HRP. After the reaction, the optical density (OD) value at 450 nm was detected by a microplate reader.

### 2.20. Statistical Analysis

The statistical software R (version 3.6.3) and GraphPad Prism 7 software (version 5.0) were used for the statistical analysis and the generation of figures. The “corrplot” R package was used to test the relationships among different immune cells. The “survival” R package was used for Kaplan-Meier curve analysis. Two-tailed unpaired *t*-tests were performed to compare the gene expression difference in two groups, and the results are presented as the mean ± SD. Each experiment had three biological replicates. *P* < 0.05 was considered to indicate a significant difference.

## 3. Results

### 3.1. Infiltrating Cell Populations in the TME Affect the Clinical Outcomes of Patients with CRC

**Figure 1:**
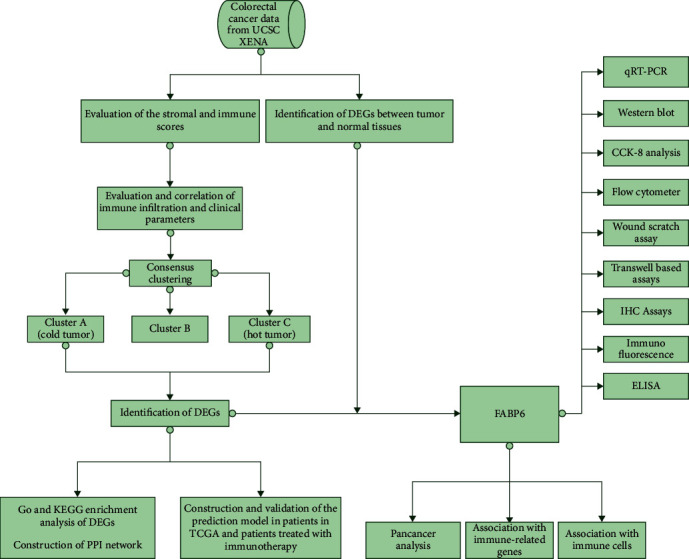
Flowchart of this study.

**Figure 2:**
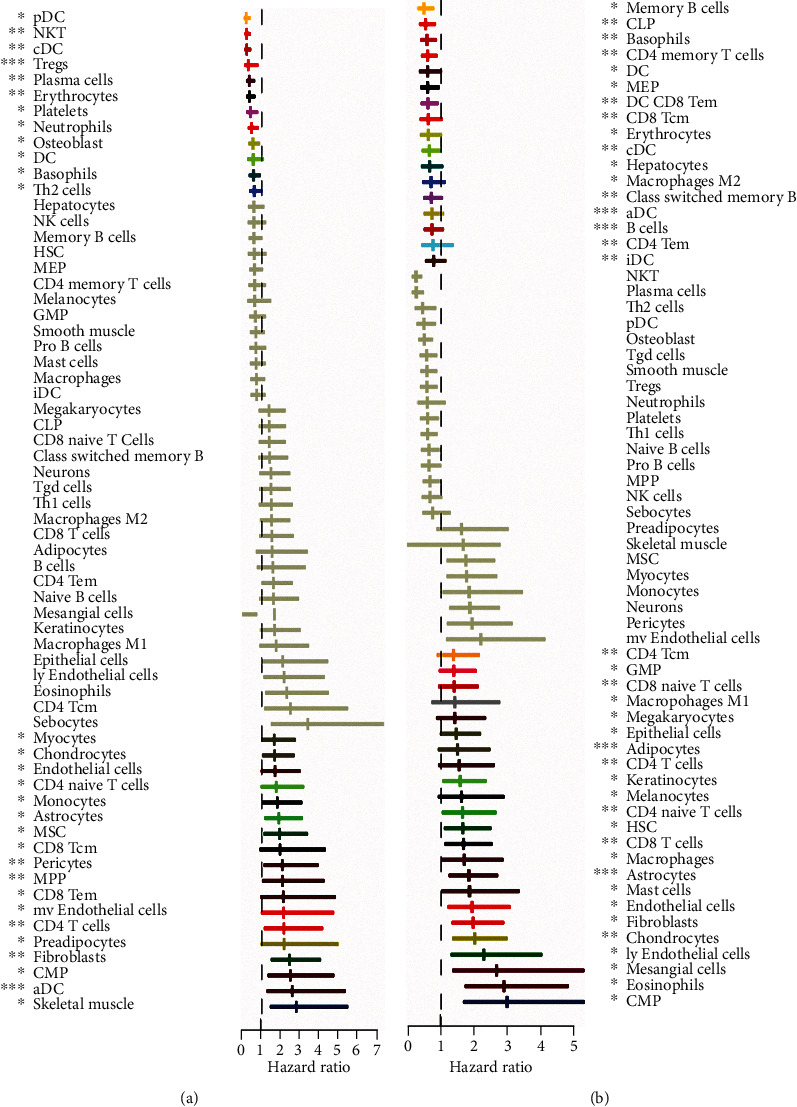
Correlations of the 64 types of cells with the survival of CRC patients. (a) Forest plot showed the associations of the 64 cell types and OS. (b) Forest plot showed the associations of the 64 cell types and PFS.

In order to reveal the immune landscape of CRC and explore promising new targets for immunotherapy, we conducted a series of bioinformatic analyses and functional experiments. The flowchart of this study is shown in Figure 1. Firstly, we calculated the scores of 64 cell types and assessed their clinical significance. We evaluated the relationships between the degrees of infiltration of 64 cell types and clinicopathological parameters (Table 1). The results revealed that the age or sex of patients hardly affected the immune infiltration of 64 different kinds of cells in the TME. Of note, infiltration of cells that perform immune functions was significantly associated with tumor status, such as TNM stage and clinical stage. The infiltration levels of CD8+ central memory T (Tcm) cells, CD4+ memory T cells, plasmacytoid dendritic cells (pDCs), and neutrophils were decreased in patients with advanced tumors, which may indicate immune suppression in these patients (Supplementary Figures 1A–1D). Next, we analyzed the correlations of the infiltration of the 64 cell types with OS and PFS. The results showed that high infiltration levels of basophils, dendritic cells (DCs), conventional dendritic cells (cDCs), and pDCs were associated with favorable OS and PFS (Figures 2(a) and 2(b), Supplementary Figures 2A and 2B). However, high proportions of astrocytes, CD4 naive T cells, CD4 T cells, chondrocytes, common myeloid progenitor (CMP) cells, endothelial cells, and fibroblasts were associated with a poor prognosis in patients (Figures 2(a) and 2(b), Supplementary Figures 3A and 3B). The above results suggest that there are large differences in the cellular composition of CRC patients with different clinicopathological parameters, and these differences are likely to be important determinants of both prognosis and treatment response, indicating that immune cells and nonimmune cells together reflect the malignant features of the tumor and the heterogeneity of the TME.

### 3.2. The Associations among Infiltrating Cells in CRC

**Figure 3:**
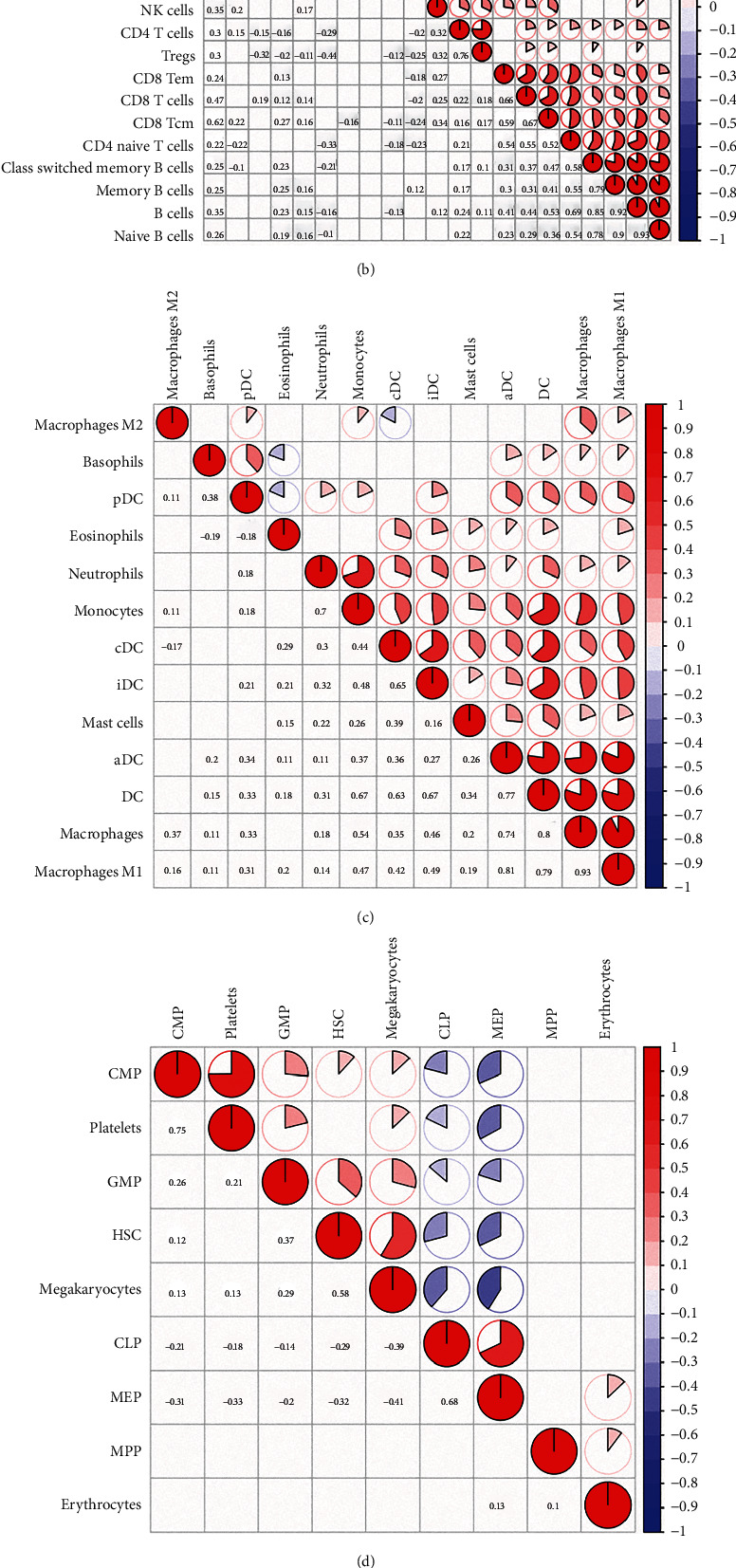
Correlations among the 64 types of cells. (a) Pie plot showed the correlations among the 64 types of cells. (b) Pie plot showed the correlations of cells in lymphoid cell groups. (c) Pie plot showed the correlations of cells in myeloid cell groups. (d) Pie plot showed the correlations of cells in stem cell groups. (e) Pie plot showed the correlations of cells in stromal cell groups. (f) Pie plot showed the correlations of cells in other cell groups.

According to the study published by Aran et al., the 64 cell types were grouped into five families, including lymphoid cells, stem cells, myeloid cells, stromal cells, and others [19]. We found that some stem/progenitor cells showed a negative association with corresponding differentiated cells; for example, megakaryocyte erythroid progenitors (MEPs) were correlated with megakaryocytes, common lymphoid progenitor (CLP) cells, CD4+ T cells, CD8+ T cells, NK cells, and B cells. On the other hand, mesenchymal stem cells (MSCs), granulocyte-macrophage progenitors (GMPs), and CMPs were positively associated with their corresponding differentiated cells. The results may indicate that the mechanism of cell differentiation for cell types is different in tumor tissues. In addition, epithelial cells showed comprehensive negative correlations with other cells in tumor tissue, indicating that they may form a barrier to prevent the infiltration of cells (Figure 3(a)). Most lymphoid cell subtypes and myeloid cells, such as CD8+ T cells, CD4+ memory T cells, DCs, and macrophages, showed strong positive correlations with each other, indicating cooperation among these cells in the immune response (Figures 3(b) and 3(c)). CLPs and MEPs were negatively correlated with other stem cells (Figure 3(d)). Among stromal cells, both smooth muscle cells and osteoblast cells were negatively correlated with other stromal cells because of their functions and distribution in tumors (Figure 3(e)). For other cell families, neurons showed a negative correlation with other cell types (Figure 3(f)). These results suggest that infiltration is the result of mutual cooperation and antagonism.

### 3.3. Characterization of the Immune Subtypes of CRC

**Figure 4:**
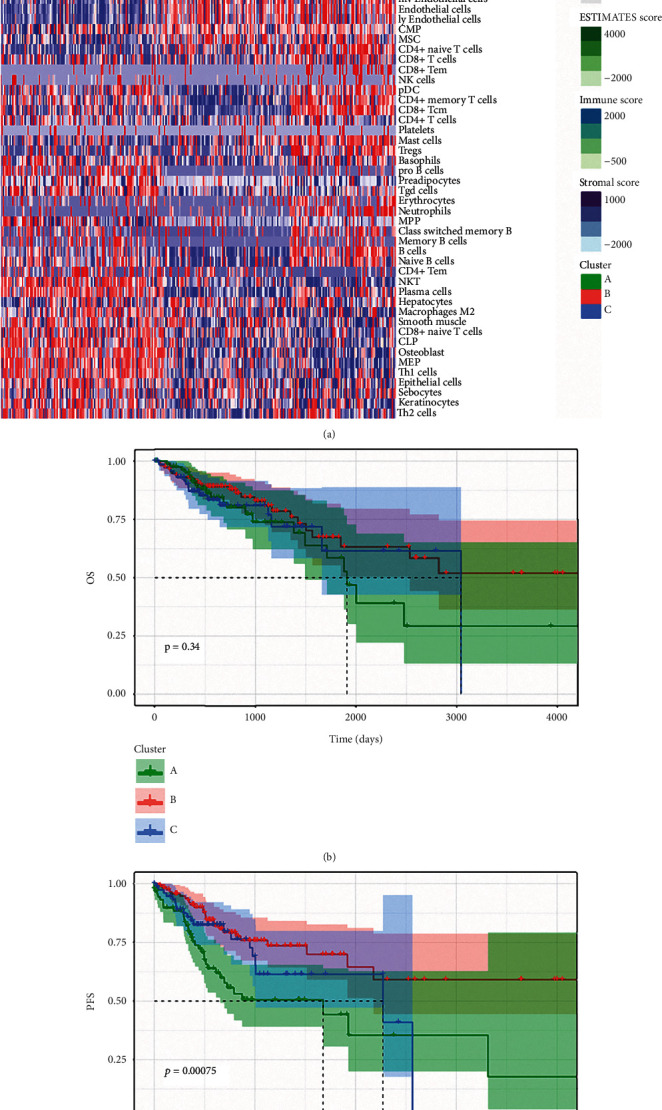
Characterization of the immune subtypes of CRC. (a) Heatmap showed the 3 subtypes of CRC. (b) OS analysis of the 3 subtypes. (c) PFS analysis of the 3 subtypes.

We performed consensus clustering of the 64 cell types in tumor tissues and divided the tumor samples into three subtypes based on the infiltration levels and infiltrating cell types (Supplementary Figures 4A and 4B). Group A contained mostly stromal cells and stem cells, with a small number of lymphoid cell subtypes and M2 macrophages. Additionally, group A had the highest tumor purity and the lowest immune score. The pattern for group B was opposite to that of group A: this group lacked stromal cells but was enriched in immune cells, including CD8+ T cells and CD4+ T cells. Group C contained mostly immune cells. Consistent with these results, group C had the highest immune score. Of note, group C was also enriched in immunosuppressive cells, including regulatory T cells (Tregs) (Figure 4(a)), suggesting that Tregs induced by tumor cells have potent immunosuppressive functions and that their roles are different from those of M2 macrophages.

To explore the survival differences among the 3 groups, we performed survival analysis. We found that group A had worse PFS than groups B and C (Figure 4(c)); although no significant difference in OS was found among the 3 clusters, group A tended to have worse OS (Figure 4(b)). Notably, despite the high immune infiltration in group C, the survival of this group was no better than that of group B, which may be caused by immunosuppressive factors such as Tregs. These results suggest that the infiltration patterns of immune cells can reflect the survival and clinical status of tumor patients.

### 3.4. Alterations in Signaling Pathways in Hot and Cold Tumors

**Figure 5:**
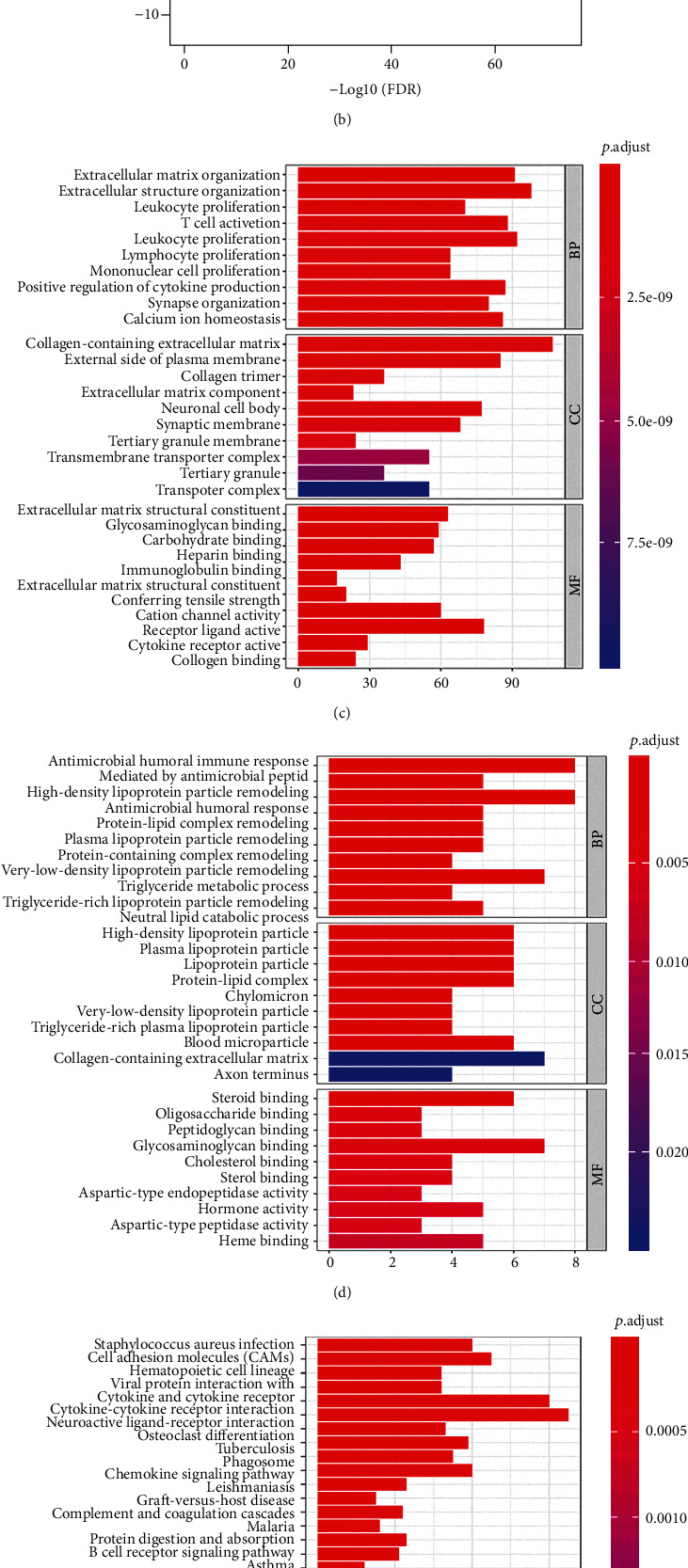
Alterations in signaling pathways in the hot and cold groups. (a) Heatmap showed the DEGs between hot and cold tumors. (b) Volcano plot showed the DEGs between hot and cold tumors. (c, d) GO enrichment analysis of hot and cold tumors. (e, f) KEGG enrichment analysis of hot and cold tumors.

To further explore the underlying mechanisms regarding immune infiltration, we redefined the samples in group C and group A as immune-high and immune-low tumors (denoted as hot and cold tumors). We first analyzed the expression of immune-related molecules in the two subtypes and observed that most immune-related genes were highly expressed in hot tumors (Supplementary Figure 5). Next, we analyzed the difference between the two groups at the transcriptional level. We found that hot and cold tumors showed different transcription patterns, as shown in the heatmap and volcano plot (Figures 5(a) and 5(b)).

We identified the DEGs between hot and cold tumors and performed GO and KEGG enrichment analyses. The enriched GO terms in hot tumors were immune-related terms, including leukocyte migration, T cell activation, and positive regulation of cytokine production, while in cold tumors, the DEGs were mostly enriched in metabolic processes, including the carboxylic acid biosynthetic process, organic acid biosynthetic process, and steroid metabolic process (Figures 5(c) and 5(d)). Pathways including neuroactive ligand-receptor interactions, cytokine-cytokine receptor interactions, and the PI3K-Akt signaling pathway were significantly enriched in hot tumors. Pathways such as cholesterol metabolism, the biosynthesis of amino acids and the metabolism of xenobiotics by cytochrome P450 were enriched in cold tumors (Figures 5(e) and 5(f)). These results suggest that metabolic reprogramming of tumor cells may suppress the immune response in the TME.

PPI analysis revealed that DEGs upregulated in hot tumors could be clustered into 4 groups, while in cold tumors, they were clustered into three groups (Supplementary Figures 6A and 6B). The top 10 DEGs with a high degree of connectivity were selected as the hub genes (Supplementary Figures 6C and 6D). The results revealed that the top 10 hub genes in hot tumors were mainly chemokines (CCL19, CCL21, and CCL13) and members of the guanine-nucleotide-binding protein- (G-protein-) coupled receptor family (CNR1, HRH3, and CHRM2). The top 10 hub genes in cold tumors mainly included apolipoprotein family members and glycoproteins (APOA2, APOA4, APOC3, and HRG). Additionally, we found that the hub genes in hot tumors, such as AMBP, C3, CCL19, HRH3, INSL5, PAH, PENK, and PYY, were associated with the survival time of patients with CRC (Supplementary Figure 6E). However, the downregulated hub genes did not significantly affect the OS of patients.

### 3.5. Construction and Validation of the Prediction Model in Patients in TCGA and Patients Treated with Immunotherapy

**Figure 6:**
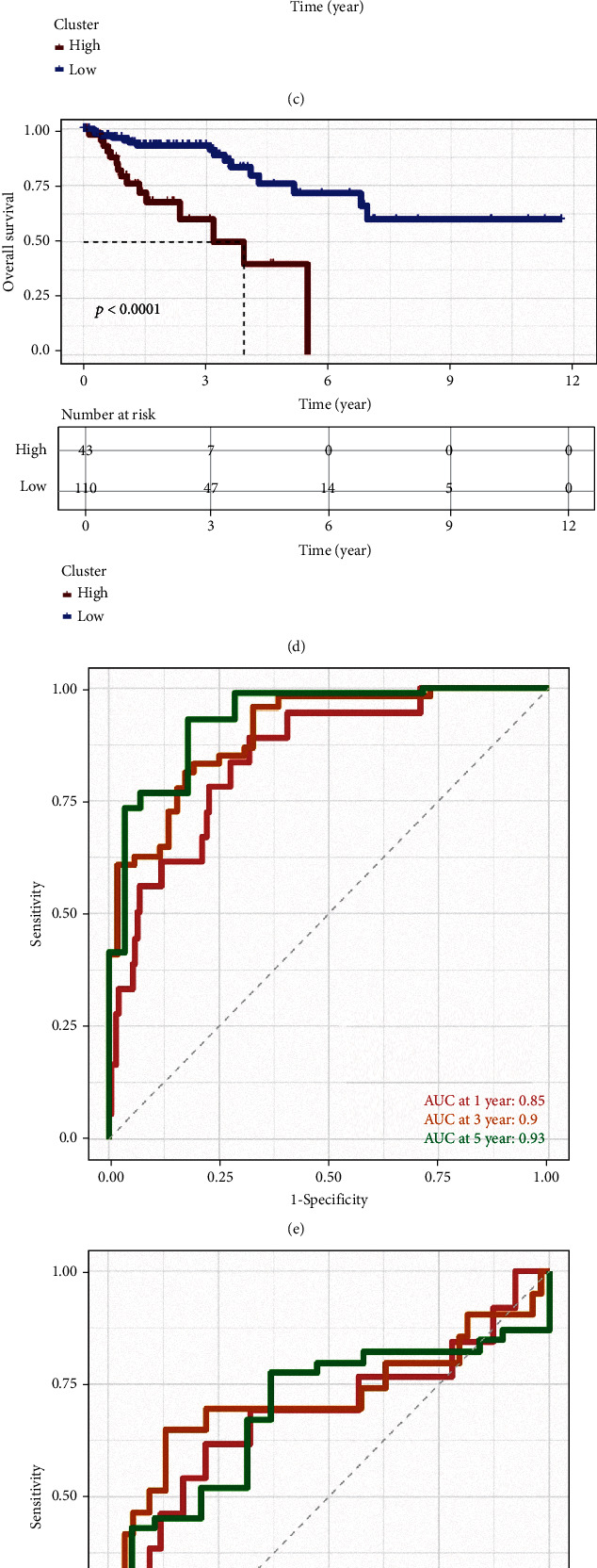
Construction and validation of the prediction model. (a, b) Correlation of the risk value and survival time in training and testing cohort. (c, d) OS analysis of high- and low-risk groups in the training and testing cohorts. (e, f) ROC curve for predicting model in the training and testing cohorts. (g, h) OS analysis of the high- and low-risk groups in the validation cohort (g: metastatic urothelial cancer patients, h: melanoma patients).

Next, we constructed a prediction model based on the DEGs. We first randomly divided the patients in TCGA into training and testing cohorts and performed univariate Cox analysis to select the survival-related genes (Figures 6(a) and 6(b)). Then, we used the LASSO regression model to further select the best combination of these genes. Finally, we used multivariate Cox regression analysis to optimize the model (Supplementary Figures 7A–7C). The prediction model contained 10 genes, and 4 of these genes were adverse prognostic factors. High-risk patients had worse survival in the training and testing cohorts (Figures 6(c) and 6(d)). The value of the receiver operating characteristic (ROC) curve in the training cohort and testing cohort revealed that this model can well predict the OS of CRC patients (Figures 6(e) and 6(f)). Of note, we also used a validation cohort of patients who received immunotherapy treatment to test the model. The results revealed that the model also performed well in predicting the OS of patients treated with immunotherapy (Figures 6(g) and 6(h)). The above results suggest that the prediction model constructed based on DEGs performed well in predicting the OS of CRC patients treated with and without immunotherapy.

### 3.6. Inhibition of FABP6 Promotes the Immunogenicity of Tumor Cells

To select potential immunotherapy targets, we identified the overlapping genes among genes upregulated in cold tumors vs. hot tumors and genes upregulated in tumor tissues vs. normal tissues (Supplementary Figure 8A). We identified FABP6 as an immunotherapy target because it ranked at the top in the two datasets. Pancancer analysis revealed that FABP6 was upregulated in most cancers, indicating its role in promoting the progression of tumor cells (Supplementary Figure 8B). Additionally, FABP6 negatively correlated with immune-related genes, including CD4, CD8A (Supplementary Figure 8C). Analysis with the Tumor IMmune Estimation Resource (TIMER) also revealed that FABP6 expression was negatively correlated with the immune infiltration of B cells, CD8+ T cells, CD4+ T cells, macrophages, neutrophils, and DCs (Supplementary Figure 8D).

**Figure 7:**
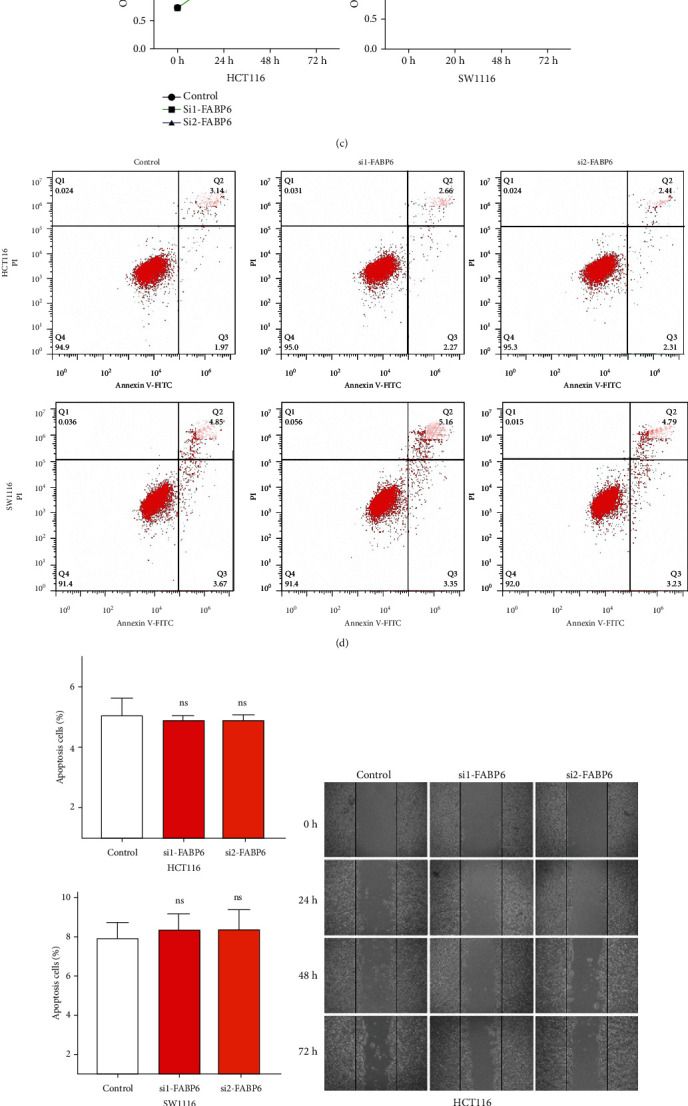
Inhibition of FABP6 does not affect proliferation, migration, and apoptosis of tumor cells. (a, b) qRT-PCR and western blot showed the efficiency of FABP6 knockdown. (c) CCK-8 analysis showed the proliferation of tumor cells with FABP6 knockdown. (d, e) Flow cytometry analysis showed the apoptosis of tumor cells with FABP6 knockdown; (d) is representative images, and (e) is the statistical analysis of the ratio (ns: not significant). (f, g) Scratch analysis showed that inhibition of FABP6 did not affect the migration of tumor cells.

To further explore the function of FABP6, we first knocked down FABP6 in two CRC cell lines, and the knockdown efficiency was confirmed at the mRNA and protein levels (Figures 7(a) and 7(b)). The results of CCK-8 analysis and flow cytometry analysis revealed that knockdown of FABP6 did not alter the proliferation or apoptosis of tumor cells (Figures 7(c)–7(e)). Scratch analysis showed that knockdown of FABP6 did not affect the migration ability of tumor cells (Figures 7(f) and 7(g)), while, interestingly, the transwell assays indicated that knockdown of FABP6 could slightly inhibit the migration and invasion compared with the control group (Supplementary Figures 9A and 9B). That may be because cell morphology changed when single cells were invading across membranes, which may affect the migration or invasive capacity in transwell assays. However, no significant difference was found between FABP6 and OS and disease-free survival (DFS) of CRC patients based on the follow-up data from TCGA database (Supplementary Figures 9C and 9D).

**Figure 8:**
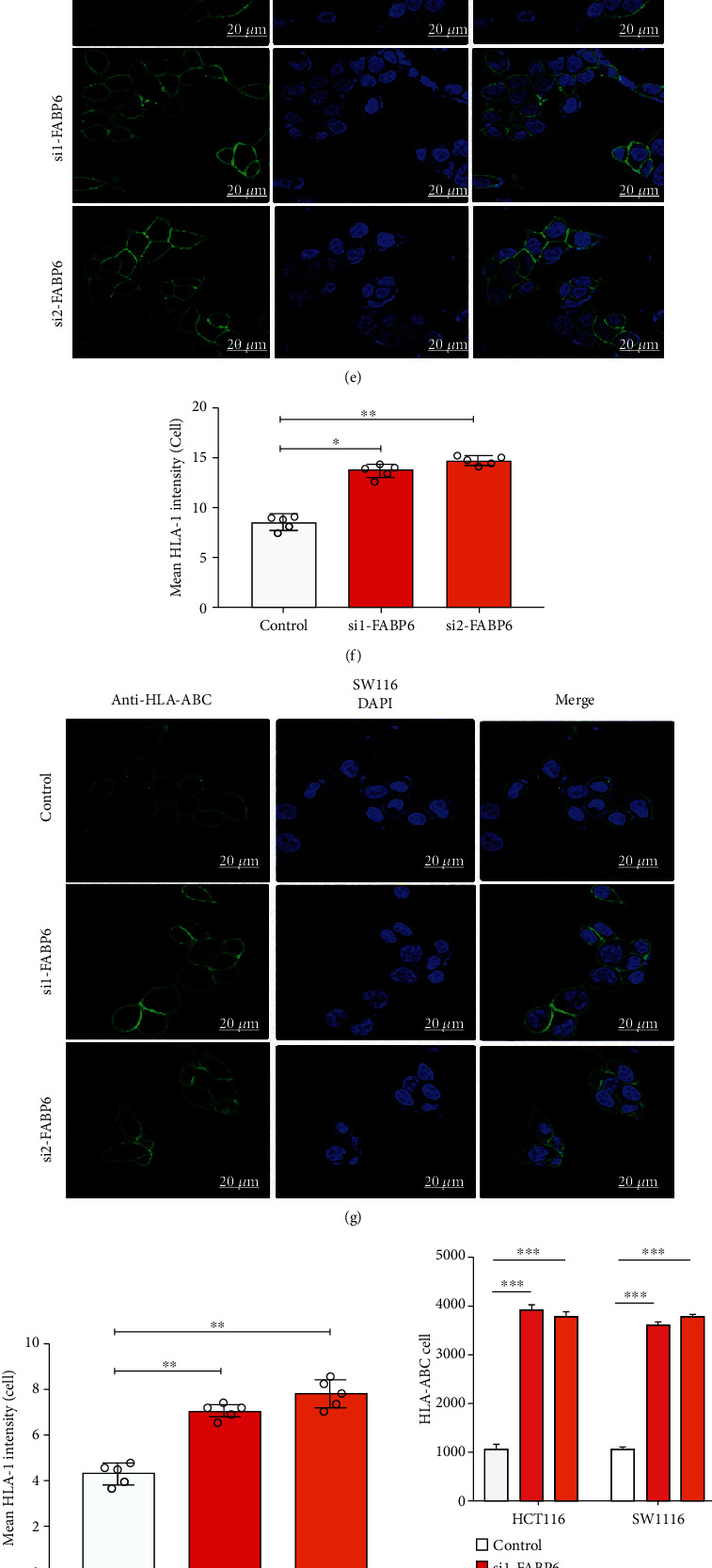
Inhibition of FABP6 promotes the immunogenicity of tumor cells. (a, b) Relative mRNA expression of immune-related genes in tumor cells. (c, d) ELISA analysis showed the secretion of CCL5 and CXCL9 in tumor cells. (e–h) Immunofluorescence analysis of HLA-A/B/C in tumor cells. (e) and (g) are representative images; (f) and (h) are statistical analysis of the intensity. (i) Flow cytometry analysis showed HLA-A/B/C expression with FABP6 knockdown in tumor cells. (j–l) Transwell analysis of CD8+ T cells; (j) is representative images, and (k) is the statistical analysis of CD8+ cell numbers. (l) IHC analysis of FABP6 and CD3 expression in clinical samples. ^∗^*P* < 0.05; ^∗∗^*P* < 0.01; ^∗∗∗^*P* < 0.001.

Notably, knockdown of FABP6 promoted the expression of immune-related genes, including major histocompatibility complex (MHC) class I and Th1-like chemokine-related genes (Figures 8(a) and 8(b)). In addition, ELISA confirmed that knockdown of FABP6 promoted the secretion of CCL5 and CXCL9 (Figures 8(c) and 8(d)). Immunofluorescence analysis revealed that knockdown of FABP6 promoted the expression of HLA-A/B/C, indicating that knockdown of FABP6 increased the immunogenicity of tumor cells (Figures 8(e)–8(h)), which was confirmed by flow cytometry (Figure 8(i), Supplementary Figure 10). To confirm whether FABP6 knockdown can increase T cell recruitment, we isolated CD8+ T cells from healthy donors and stimulated them with CD3/CD28 beads. The conditioned medium from tumor cells was collected after the knockdown of FABP6 for 48 h and added to the lower chamber. The results revealed that knockdown of FABP6 promoted the recruitment of CD8+ T cells (Figures 8(j) and 8(k)). Consistent with these results, analysis of clinical samples also revealed that the intensity of FABP6 staining was negatively correlated with the intensity of CD3 staining (Figure 8(l)). These results suggest that FABP6 may serve as a potential target for immunotherapy.

## 4. Discussion

In recent decades, tumor infiltration research has often focused on immune cell infiltration, especially infiltrating T lymphocytes, and has achieved remarkable results. However, increasing evidence has proven that other cell types, such as stromal cells, stem cells, and even keratinocytes, also play crucial roles in tumor progression and cell interactions [20–22]. Here, we described the comprehensive landscape of tumor infiltration patterns in CRC and explored potential targets for immunotherapy.

In this research, we analyzed the distribution and level of 64 human cell types in the TME and the correlations of those cell types with the pathological characteristics of patients. As we expected, the infiltration levels of some kinds of lymphoid cells, such as CD4+ memory T cells and CD8+ Tcm cells, were associated with favorable clinical features and pathological diagnoses of patients. pDCs are a multifunctional population known for their specialized ability to produce and secrete a large amount of type I interferons (IFNs) [23]. Several studies have shown that pDCs can be recruited to the TME and produce type 1 IFNs to promote antitumor immunity and that they can also present antigens to activate CD4 T cells or CD8 T cells through cross-presentation [24, 25]. We found that the infiltration of pDCs was lower in advanced tumors than in early-stage tumors, which may indicate immune suppression in patients with advanced tumors. Surprisingly, the number of neuronal cells was significantly associated with the extent of distant metastasis and the tumor clinical stage; however, the role of neuronal cells in tumors is still unclear.

The highly complex and heterogeneous system of a tumor contains not only malignant cells but also interacting cells from the host, such as endothelial cells, stromal fibroblasts, and a variety of immune cells that control tumor growth and invasion [26]. The immune phenotypes of tumors are dependent on the interaction between tumor cells and cells infiltrating the microenvironment. Tumor cells coexist with immune cells and nonimmune cells and establish subtle interactions with them that determine the tumor's immune phenotypes. Here, we revealed the complicated synergistic and antagonistic relationships between different infiltrating immune cells in CRC based on the distribution of those cells in the TME. Macrophages are a heterogeneous population of myeloid cells of the innate immune system and are particularly active in inflammation and infection. Activated type 1 (M1) and alternatively activated type 2 (M2) are two major polarization states of macrophages. Among tumor-infiltrating immune cells, macrophages are the most abundant and are called tumor-associated macrophages (TAMs). M1 macrophages are characterized by the production of proinflammatory cytokines such as TNF-*α*, IL-1*β*, IL-6, and IL-12 [27]. In contrast to M1 macrophages, M2 macrophages are immunosuppressive and favor angiogenesis and tissue repair. However, we found that macrophages (M0), M1 macrophages, and M2 macrophages are positively correlated with each other in tumor infiltration, which means that these cells all have the same infiltration pattern (low or high) in the TME of a given sample. The same phenomenon was also observed for myeloid cells and stromal cells. The findings provide a new perspective for understanding tumor infiltration.

Immune contexture was adopted to refer to the combination of immune variables associated with the nature, density, immune functional orientation, and immune cell distribution within the tumor. Rather than the traditional classification of tumors, tumors can be classified as hot tumors (high infiltration levels) or cold tumors (low infiltration levels) according to the immune infiltration level. The terms hot and cold are routinely used to refer to T cell-infiltrated/inflamed versus noninfiltrated/noninflamed tumors, respectively, accurately reflecting the higher and lower immune score categories. The characteristics of hot tumors are the presence of numerous tumor-infiltrating lymphocytes (TILs), the expression of antiprogrammed death-ligand 1 (PD-L1) on tumor-associated immune cells, and a strong antitumor immune response. Conversely, cold tumors indicate poor infiltration of immune cells, low expression of neoantigens, and low expression of antigen presentation machinery markers such as MHC class I [28]. This classification method of tumors reflects the outcome of the tremendously complex interplay between the tumor and the immune system and is beneficial for guiding treatment selection. Based on this classification, we divided the tumor samples into three groups. As we expected, patients with cold tumors had worse OS and PFS than those with hot tumors. Interestingly, patients in group B had the best OS and PFS among the three groups of patients. However, we did not further analyze group B. The analysis of this study focused on hot tumors and cold tumors.

Metabolism in tumor issues is usually altered compared with that in normal tissues because of physical pressure, oxidative stress, nutrient deprivation and competition, hypoxia, and immune surveillance in the TME. GO and KEGG analyses of DEGs between hot tumors and cold tumors revealed changes in metabolism. We found that the downregulated DEGs were significantly enriched in biosynthetic and bioenergetic metabolic processes, such as carboxylic acid biosynthetic process, organic acid biosynthetic process, steroid metabolic process, and lipid catabolic process. Corresponding to the GO analysis, KEGG analysis showed that the downregulated DEGs were enriched in the cholesterol metabolism process, biosynthesis of amino acids process, and metabolism process of xenobiotics by cytochrome P450. The results indicated that the metabolism of cold tumors was suppressed compared with that of hot tumors. The suppression of biosynthetic and bioenergetic metabolic processes in cold tumors may explain the worse OS and PFS of patients with these tumors because nutrient deficiency can impair the activity of immune cells involved in tumor destruction [29, 30].

FABP6 is a member of the fatty acid-binding protein (FABP) family, which is a class of chaperones that participate in the uptake, transport, storage, and metabolism of cellular fatty acids; cellular signaling; and gene transcription regulation [31–33]. Ohmachi et al. reported that FABP6 is overexpressed in CRC patients compared with healthy donors and leads to an increased risk of colon cancer [34]. In our study, FABP6 was selected for functional study because it ranked at the top in both cold tumor vs. hot tumor group and tumor tissue vs. normal tissue group. Firstly, the pancancer analysis showed FABP6 was also upregulated in most cancers, indicating that FABP6 may act as a protumor factor in cancers. Then, we found that FABP6 expression was negatively correlated with immune-related genes and the immune infiltration of CD8+ T cells and CD4+ T cells. In in vitro experiments, the proliferation, apoptosis, and migration of tumor cells were hardly altered after suppression of FABP6 expression, even though the transwell assays showed a slightly decreasing trend of migration and invasion. Inconsistent with our findings, overexpression of FABP6 in DLD-1 cells decreased the proliferation of CRC tumor cells [34]. The different tumor cell lines used in the two studies and the time of analyzing proliferation may contribute to the difference. In addition, the baseline expression of FABP6 in the tumor cell lines also affects the function of knockdown and overexpression. Sequentially, our study showed that the suppression of FABP6 using siRNA enhanced the immunogenicity of tumor cells. For example, the expression of CCL4, CCL5, CXCL9, HLAA, HLAB, HLAC, TAP1, and TAP2 was increased after FABP6 was suppressed in the CRC lines HCT116 and SW1116, indicating that the chemotaxis of various immune cells and antigen presentation of tumor cells were strongly enhanced. In addition, this study revealed that the knockdown of FABP6 can increase the recruitment of CD8+ T cells, and the FABP6 staining was negatively correlated with the intensity of CD3 staining. Considering the above results and the restricted expression of FABP6 in normal small intestine tissue, FABP6 may be a candidate biomarker for diagnosing CRC.

However, there are also some limitations in our study. First, we did not validate the prediction model in an external dataset. Second, for lacking of enough follow-up data, we used the survival data from TCGA database to explore the prognostic roles of FABP6 instead of clinical patients, which should be further studied in the near future. Third, we did not explore the mechanisms on how FABP6 regulates CCL5 and CXCL9 expression. At last, the role of FABP6 should be more thoroughly explored in in vivo experiments.

## 5. Conclusion

Our study revealed that not only immune cells but also nonimmune cells contributed to the complicated immune infiltration pattern of tumors and reflected the status of tumor progression. We redefined tumors as hot tumors or cold tumors based on the infiltration of 64 types of human cells into the TME and analyzed the difference in checkpoint and immune response-associated molecules between the two groups. In particular, FABP6 expression was negatively correlated with immune infiltration, and knockdown of FABP6 not only increased MHC class 1 expression but also promoted immune-related chemokine secretion, resulting in the recruitment of CD8+ T cells. In summary, this study provides a comprehensive landscape of the immune infiltration patterns of CRC and presents FABP6 as a potential immunotherapy target.

## Figures and Tables

**Table 1:** Association of infiltrating cells with clinical parameters of CRC.

Cell type	Cell	Age	Gender	T	M	N	Stage
Lymphoid	B cells	0.4331	0.9229	0.3311	0.0711	0.2753	0.3216
Lymphoid	CD4+ memory T cells	0.1685	0.3341	0.0105	0.0005	0.0003	<0.0001
Lymphoid	CD4+ naive T cells	0.2715	0.1667	0.7178	0.5379	0.7862	0.7756
Lymphoid	CD4+ T cells	0.0203	0.7075	0.0122	0.4617	0.5951	0.0364
Lymphoid	CD4+ Tcm	0.9147	0.3209	0.7854	0.2878	0.3610	0.3814
Lymphoid	CD4+ Tem	0.1952	0.8937	0.7658	0.7544	0.6051	0.9284
Lymphoid	CD8+ naive T cells	0.4654	0.2640	0.3159	0.8947	0.9549	0.9656
Lymphoid	CD8+ T cells	0.5679	0.4823	0.2793	0.2707	0.7788	0.4960
Lymphoid	CD8+ Tcm	0.2648	0.9934	0.0901	0.0079	0.0010	0.0008
Lymphoid	CD8+ Tem	0.1853	0.9892	0.5123	0.8273	0.7474	0.5987
Lymphoid	Class-switched memory B cells	0.2338	0.3230	0.4120	0.0678	0.7427	0.3307
Lymphoid	Memory B cells	0.2845	0.1776	0.0388	0.7745	0.1823	0.2642
Lymphoid	Naive B cells	0.9631	0.7150	0.2614	0.1141	0.0277	0.2710
Lymphoid	NK cells	0.4478	0.5688	0.8202	0.0225	0.2271	0.0979
Lymphoid	NKT	0.5926	0.0208	0.3688	0.4971	0.7148	0.4856
Lymphoid	Plasma cells	0.0380	0.1735	0.2067	0.0428	0.0500	0.1081
Lymphoid	Pro B cells	0.9577	0.2095	0.8183	0.4485	0.1552	0.2736
Lymphoid	Tgd cells	0.0193	0.5322	0.1506	0.9267	0.4513	0.5512
Lymphoid	Th1 cells	0.8939	0.4146	0.2188	0.5725	0.0215	0.2738
Lymphoid	Th2 cells	0.9263	0.6690	0.0764	0.0297	0.0004	0.0019
Lymphoid	Tregs	0.0623	0.6896	0.1345	0.0300	0.1210	0.1098
Myeloid	aDC	0.5990	0.4334	0.7332	0.0518	0.4975	0.2990
Myeloid	Basophils	0.0109	0.1752	0.1524	0.2680	0.0331	0.2303
Myeloid	cDC	0.5785	0.2220	0.5001	0.0171	0.1049	0.0305
Myeloid	DC	0.5328	0.6729	0.2501	0.0120	0.3258	0.1478
Myeloid	Eosinophils	0.4899	0.9710	0.0211	0.0159	0.0000	0.0000
Myeloid	iDC	0.9023	0.4082	0.2117	0.3823	0.5198	0.6304
Myeloid	Macrophages	0.3038	0.8727	0.4117	0.0431	0.0582	0.0346
Myeloid	Macrophages M1	0.5832	0.6410	0.3843	0.0975	0.2013	0.1253
Myeloid	Macrophages M2	0.4376	0.2932	0.8355	0.7953	0.5147	0.8442
Myeloid	Mast cells	0.4878	0.5348	0.8239	0.5542	0.4184	0.5966
Myeloid	Monocytes	0.7594	0.9413	0.1399	0.7711	0.7740	0.7820
Myeloid	Neutrophils	0.5715	0.9394	0.0314	0.0307	0.0277	0.0300
Myeloid	pDC	0.4878	0.1857	0.0081	0.0022	<0.0001	0.0001
Other	Astrocytes	0.5402	0.4300	0.0230	0.4676	0.0263	0.1121
Other	Epithelial cells	0.2242	0.8283	0.8236	0.0731	0.5693	0.3794
Other	Hepatocytes	0.3483	0.3790	0.9270	0.7129	0.3026	0.8988
Other	Keratinocytes	0.2701	0.2621	0.2829	0.2216	0.3601	0.2688
Other	Melanocytes	0.5312	0.2753	0.0215	0.7319	0.2784	0.0656
Other	Mesangial cells	0.2431	0.5943	0.0191	0.5577	0.0144	0.1075
Other	Neurons	0.2688	0.1050	0.0351	0.0079	0.0013	0.0070
Other	Sebocytes	0.1608	0.4085	0.4273	0.2669	0.3606	0.7099
Stem	CLP	0.5245	0.5893	0.1274	0.5156	0.2423	0.4070
Stem	CMP	0.2126	0.2562	0.5991	0.6218	0.6962	0.4656
Stem	Erythrocytes	0.2366	0.0489	0.2673	0.0985	0.0274	0.1135
Stem	GMP	0.7642	0.3148	0.9758	0.6856	0.9252	0.9519
Stem	HSC	0.0641	0.3273	0.2119	0.1738	0.0063	0.1430
Stem	Megakaryocytes	0.2582	0.2321	0.9708	0.0599	0.4856	0.3181
Stem	MEP	0.3958	0.5632	0.1351	0.6039	0.0952	0.2151
Stem	MPP	0.4012	0.2820	0.6813	0.6082	0.0208	0.0942
Stem	Platelets	0.8406	0.8540	0.0003	0.0539	0.4471	0.1073
Stromal	Adipocytes	0.7321	0.7567	0.7096	0.6524	0.1163	0.5702
Stromal	Chondrocytes	0.6528	0.3439	0.0222	0.5252	0.0020	0.0988
Stromal	Endothelial cells	0.0589	0.5077	0.1200	0.1627	0.0786	0.1316
Stromal	Fibroblasts	0.3939	0.5525	0.0354	0.0821	0.0408	0.0606
Stromal	ly endothelial cells	0.0443	0.3699	0.0803	0.0998	0.0937	0.0949
Stromal	MSC	0.3781	0.7674	0.0118	0.4355	0.0404	0.0080
Stromal	mv endothelial cells	0.1498	0.6796	0.0380	0.4166	0.1518	0.1223
Stromal	Myocytes	0.2317	0.5450	0.5625	0.4406	0.4189	0.7606
Stromal	Osteoblast	0.4394	0.9622	0.0709	0.2799	0.0162	0.1301
Stromal	Pericytes	0.3220	0.6211	0.1709	0.2183	0.0467	0.0505
Stromal	Preadipocytes	0.3210	0.0901	0.4713	0.9351	0.0603	0.4551
Stromal	Skeletal muscle	0.0396	0.8586	0.5951	0.0361	0.6315	0.0951
Stromal	Smooth muscle	0.8471	0.8517	0.1237	0.9616	0.1869	0.0335

## Data Availability

The data support the findings of this study are available from the corresponding author upon reasonable request.

## Abbreviations

CRC: Colorectal cancer
TME: Tumor microenvironment
ECM: Extracellular matrix
NGS: Next-generation sequencing
TNM: Tumor-node-metastasis
OS: Overall survival
CAFs: Cancer-associated fibroblasts
EMT: Epithelial-mesenchymal transition
PFS: Progression-free survival
DEGs: Differentially expressed genes
FDR: False discovery rate
GO: Gene Ontology
KEGG: Kyoto Encyclopedia of Genes and Genomes
BPs: Biological processes
MFs: Molecular functions
CCs: Cellular components
PPI: Protein-protein interaction
STRING: Search Tool for the Retrieval of Interacting Genes/Proteins
MCODE: Molecular COmplex Detection
TCGA: The Cancer Genome Atlas
LASSO: Least absolute shrinkage and selection operator
IHC: Immunohistochemistry
TBS: Tris-buffered saline
siRNA: Small interfering RNA
NC: Negative control
qRT-PCR: Quantitative real-time PCR
GAPDH: Glyceraldehyde-3-phosphate dehydrogenase
HRP: Horseradish peroxidase
CCK-8: Cell Counting Kit 8
OD: Optical density
pDCs: Plasmacytoid dendritic cells
cDCs: Conventional dendritic cells
CMP: Common myeloid progenitor
MEPs: Megakaryocyte erythroid progenitors
GMPs: Granulocyte-macrophage progenitors
ROC: Receiver operating characteristic
TIMER: Tumor IMmune Estimation Resource
MHC: Major histocompatibility complex
IFNs: Type I interferons
TAMs: Tumor-associated macrophages
TILs: Tumor-infiltrating lymphocytes
PD-L1: Programmed death-ligand 1
FABP: Fatty acid-binding protein.

## Supplementary Materials

Supplementary 1 Supplementary Figure 1: associations of infiltration of immune cells with the clinical parameters. (A–D) Representative boxplots showed the correlations of CD8+ Tcm cells, memory CD4+ T cells, pDCs, and neutrophils with clinical parameters.

Supplementary 2 Supplementary Figure 2: infiltrating cells positively correlated with survival. (A) Immune cells positively correlated with OS. (B) Immune cells positively correlated with PFS.

Supplementary 3 Supplementary Figure 3: infiltrating cells negatively correlated with survival. (A) Immune cells negatively correlated with OS. (B) Immune cells negatively correlated with PFS.

Supplementary 4 Supplementary Figure 4: consensus clustering of CRC. (A) The delta program showed the correlation of clusters and the area under the cumulative distribution function (CDF) curve. (B) Heatmap showed the selected clusters.

Supplementary 5 Supplementary Figure 5: expression of immune-related genes in hot and cold tumors.

Supplementary 6 Supplementary Figure 6: identification of hub genes. (A, B) PPI network of DEGs in hot and cold tumors; A: hot, B: cold. (C, D) Top 10 hub genes and connected genes in hot and cold tumors; C: hot, D: cold. (E) Survival analysis of hub genes and OS.

Supplementary 7 Supplementary Figure 7: identification of prognosis-related genes. (A, B) LASSO and partial likelihood deviance coefficient profiles of survival-related genes. (C) Forest plots showing the hazard ratios (HRs) of selected genes from the multivariate Cox regression analysis.

Supplementary 8 Supplementary Figure 8: identification of FABP6 and assessment of its correlation with immune infiltration in CRC. (A) Venn diagram showed the intersected upregulated genes between cold tumors vs. hot tumors and tumor tissues vs. normal tissues groups. (B) Pancancer analysis showed the expression of FABP6 in tumor and normal tissue. (C) Correlation of FABP6 and immune-related genes. (D) Correlation of FABP6 and immune cell infiltration.

Supplementary 9 Supplementary Figure 9: (A, B) transwell assays showed that FABP6 knockdown could slightly inhibit the migration and invasion abilities of tumor cells (A: migration, B: invasion). (C, D) Survival analysis between FABP6 and OS and DFS of CRC patients.

Supplementary 10 Supplementary Figure 10: mean fluorescence intensity HLA-A/B/C expression with FABP6 knockdown in groups of control, si1-FABP6, and si2-FABP6.

Supplementary 11 Supplementary Table 1: primers used in this study.
