# Supplementary figures and images for: FABP6 Expression Correlates with Immune Infiltration and Immunogenicity in Colorectal Cancer Cells
Source: J Immunol Res. 2022 Aug 17;2022:3129765. doi: 10.1155/2022/3129765 (PMC9403257; doi:10.1155/2022/3129765)

**A**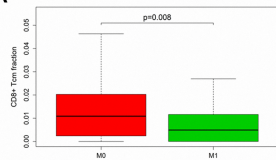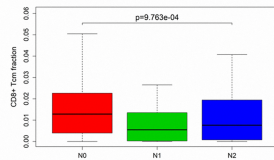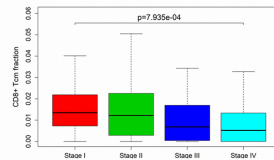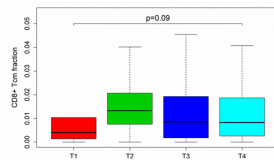**B**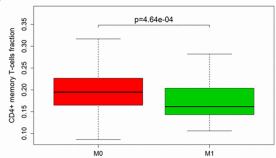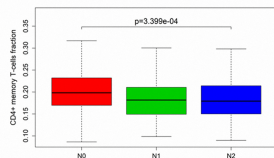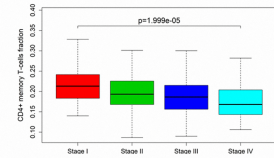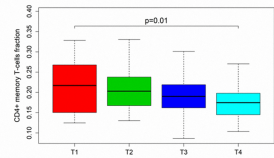**C**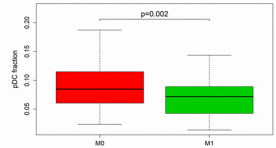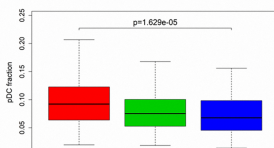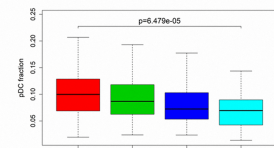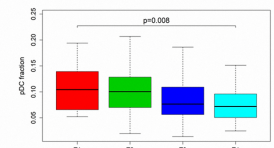**D**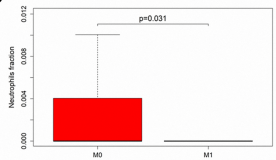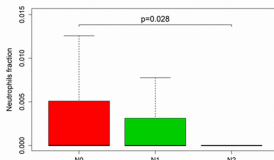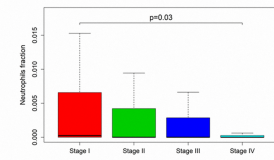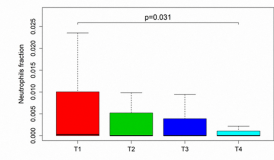

Supplement: Supplementary 1 — Supplementary Figure 1: associations of infiltration of immune cells with the clinical parameters. (A–D) Representative boxplots showed the correlations of CD8+ Tcm cells, memory CD4+ T cells, pDCs, and neutrophils with clinical parameters. [file 3129765.f1.pdf]

**A**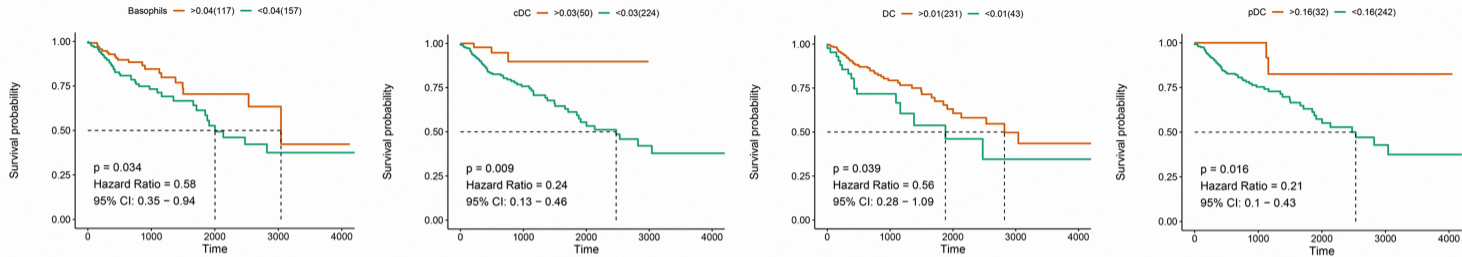**B**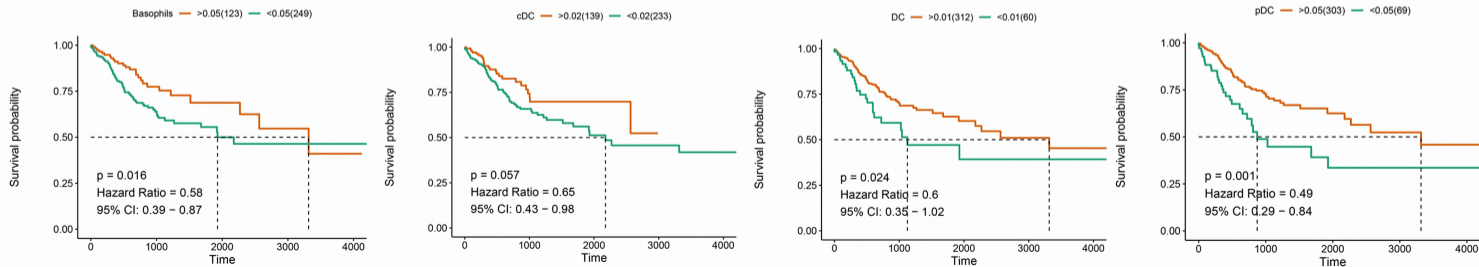

Supplement: Supplementary 2 — Supplementary Figure 2: infiltrating cells positively correlated with survival. (A) Immune cells positively correlated with OS. (B) Immune cells positively correlated with PFS. [file 3129765.f2.pdf]

A

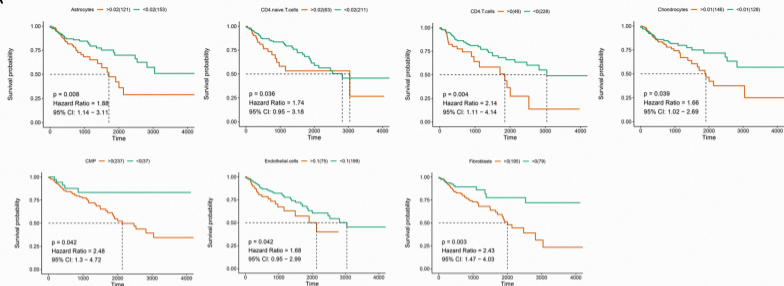

B

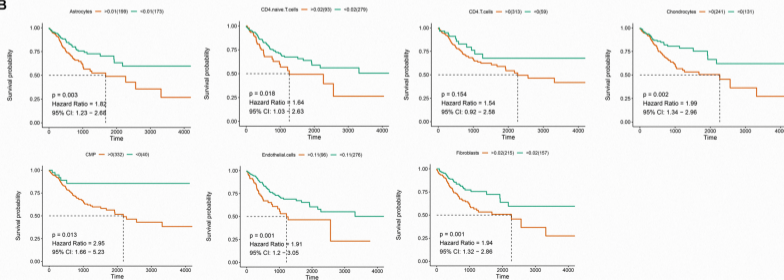

Supplement: Supplementary 3 — Supplementary Figure 3: infiltrating cells negatively correlated with survival. (A) Immune cells negatively correlated with OS. (B) Immune cells negatively correlated with PFS. [file 3129765.f3.pdf]

**A**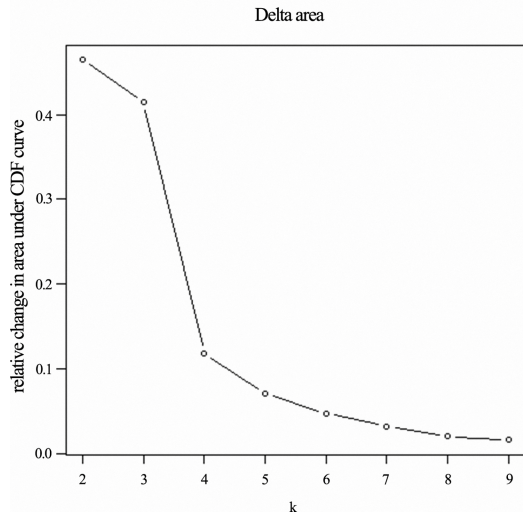**B**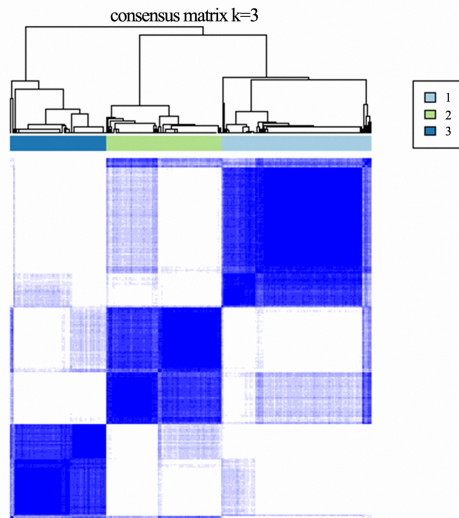

Supplement: Supplementary 4 — Supplementary Figure 4: consensus clustering of CRC. (A) The delta program showed the correlation of clusters and the area under the cumulative distribution function (CDF) curve. (B) Heatmap showed the selected clusters. [file 3129765.f4.pdf]

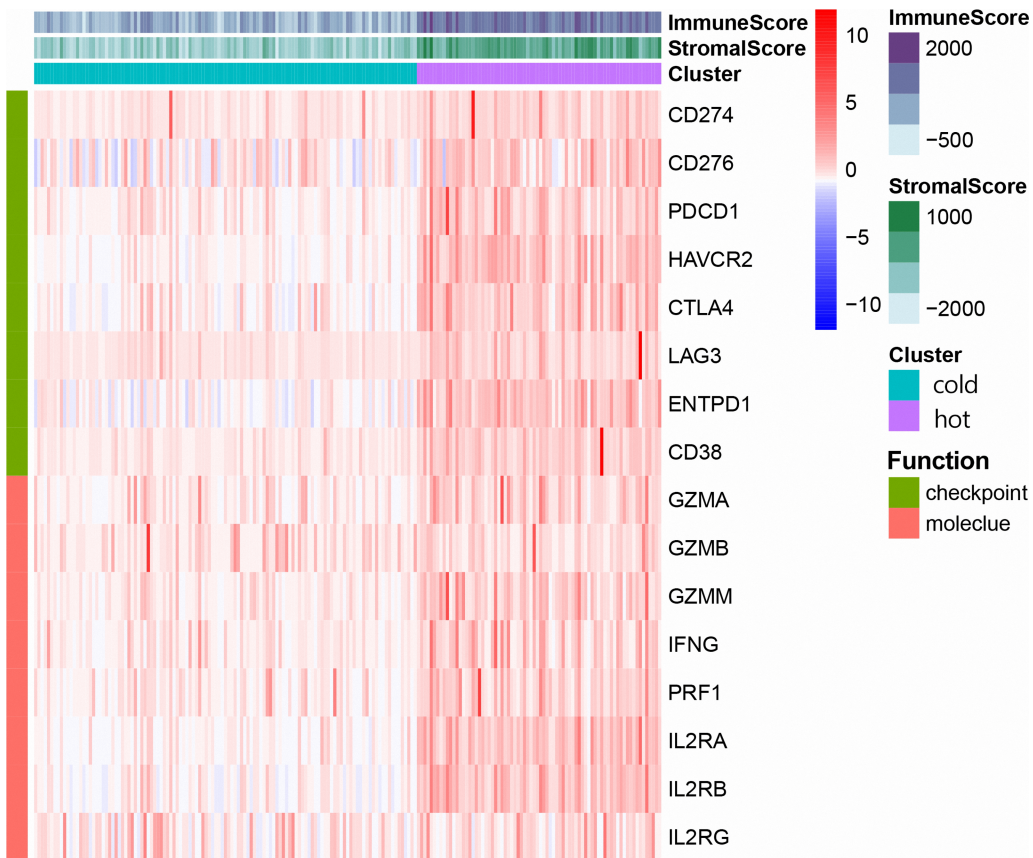

Supplement: Supplementary 5 — Supplementary Figure 5: expression of immune-related genes in hot and cold tumors. [file 3129765.f5.pdf]

**A**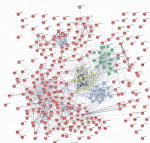**B**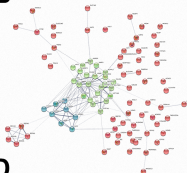**C**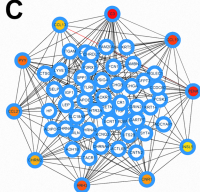**D**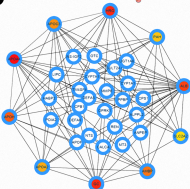**E**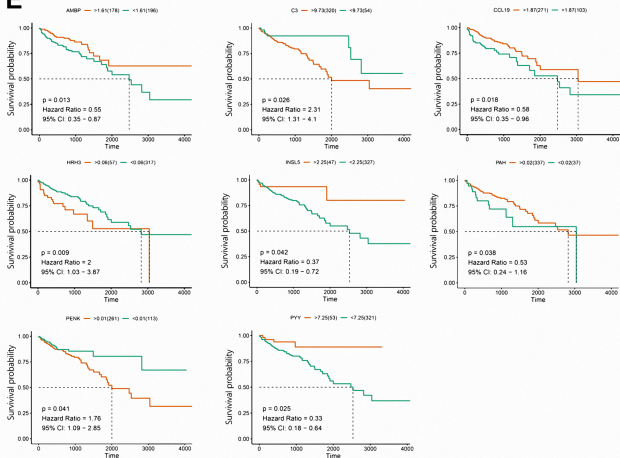

Supplement: Supplementary 6 — Supplementary Figure 6: identification of hub genes. (A, B) PPI network of DEGs in hot and cold tumors; A: hot, B: cold. (C, D) Top 10 hub genes and connected genes in hot and cold tumors; C: hot, D: cold. (E) Survival analysis of hub genes and OS. [file 3129765.f6.pdf]

A

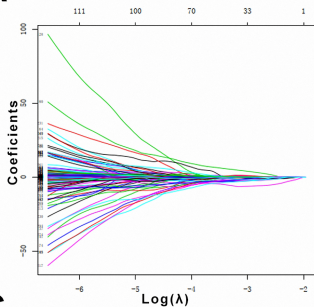

B

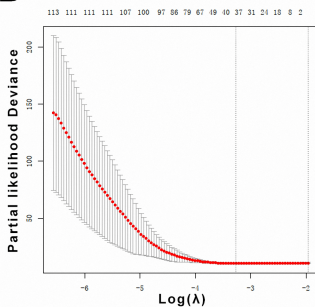

C

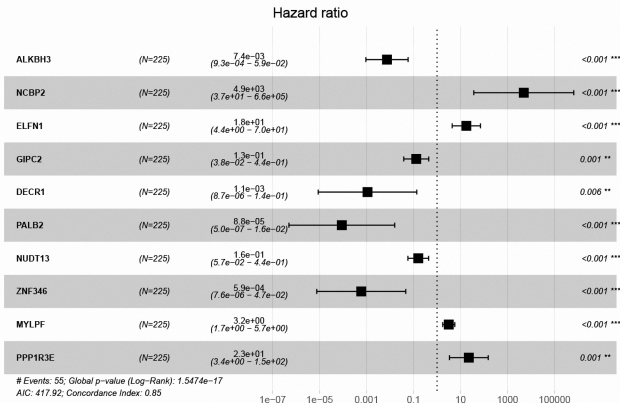

Supplement: Supplementary 7 — Supplementary Figure 7: identification of prognosis-related genes. (A, B) LASSO and partial likelihood deviance coefficient profiles of survival-related genes. (C) Forest plots showing the hazard ratios (HRs) of selected genes from the multivariate Cox regression analysis. [file 3129765.f7.pdf]

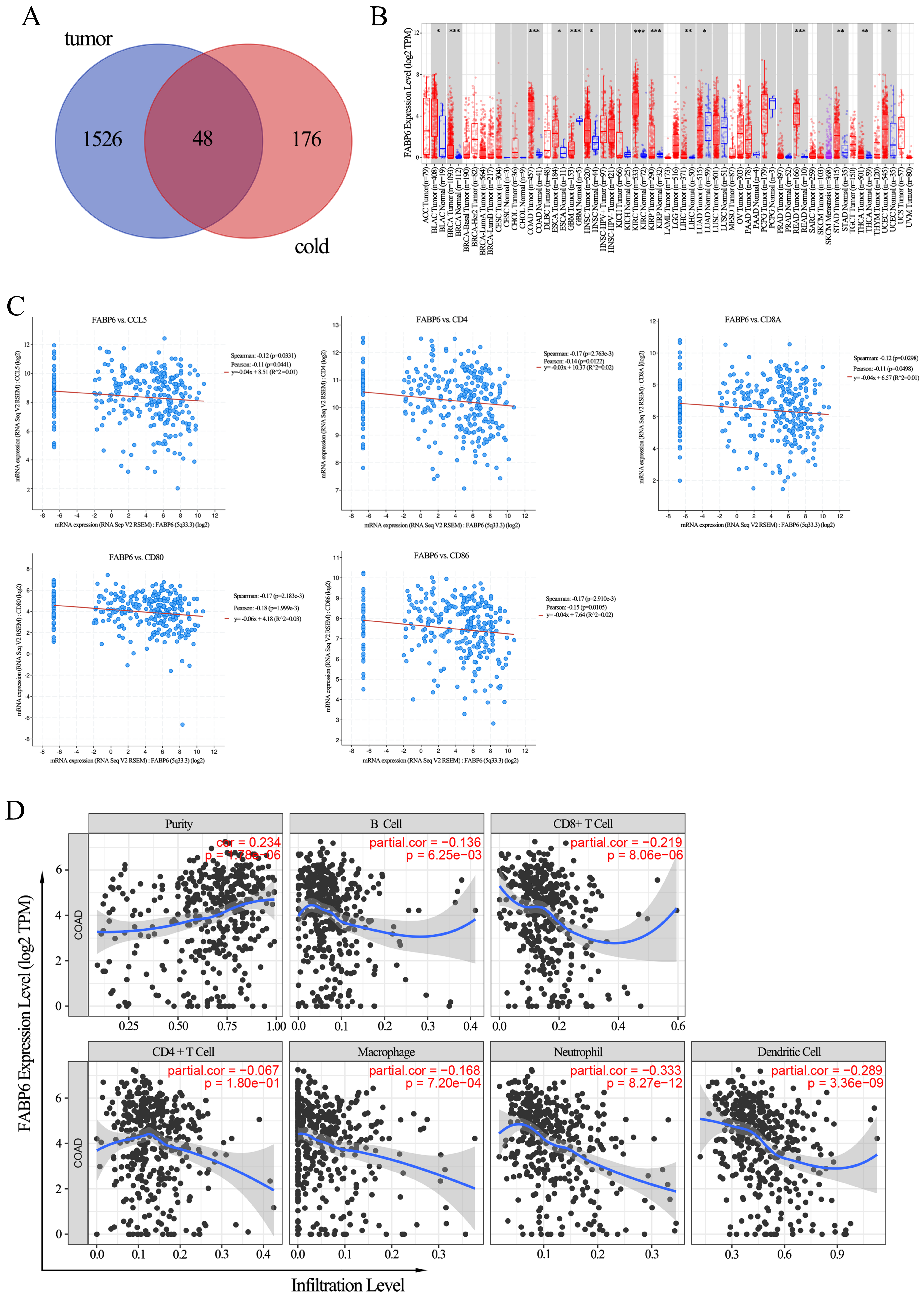

Supplement: Supplementary 8 — Supplementary Figure 8: identification of FABP6 and assessment of its correlation with immune infiltration in CRC. (A) Venn diagram showed the intersected upregulated genes between cold tumors vs. hot tumors and tumor tissues vs. normal tissues groups. (B) Pancancer analysis showed the expression of FABP6 in tumor and normal tissue. (C) Correlation of FABP6 and immune-related genes. (D) Correlation of FABP6 and immune cell infiltration. [file 3129765.f8.pdf]

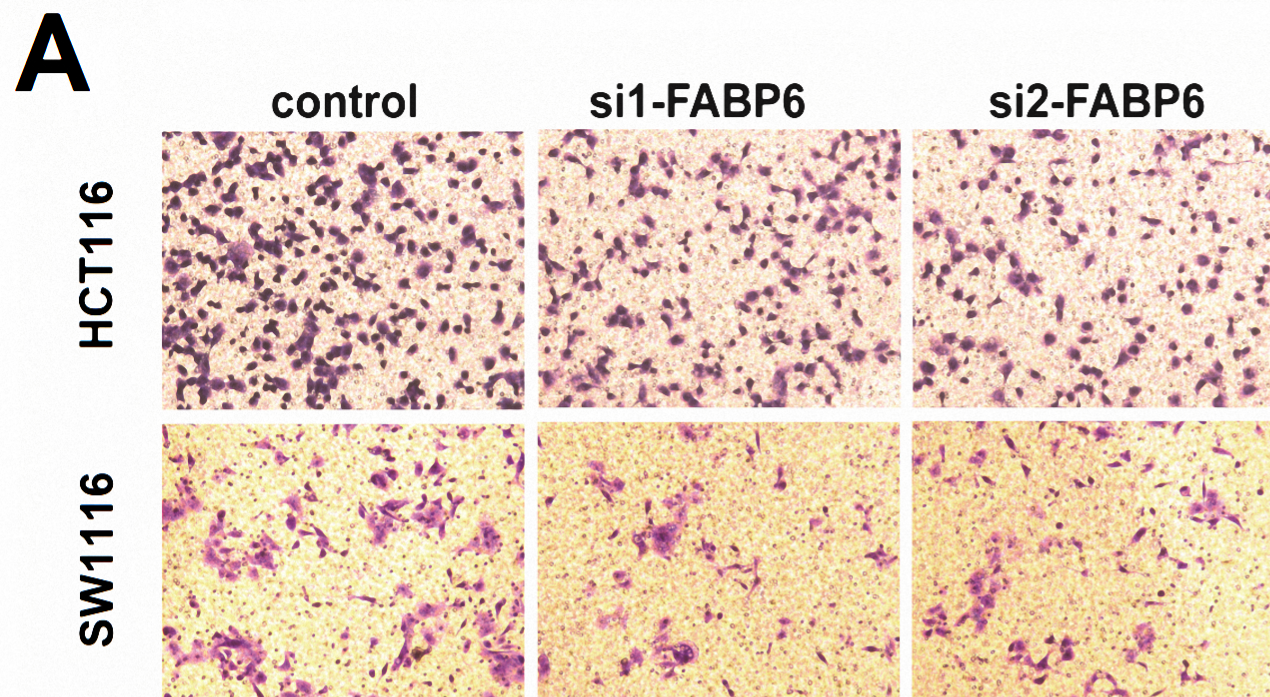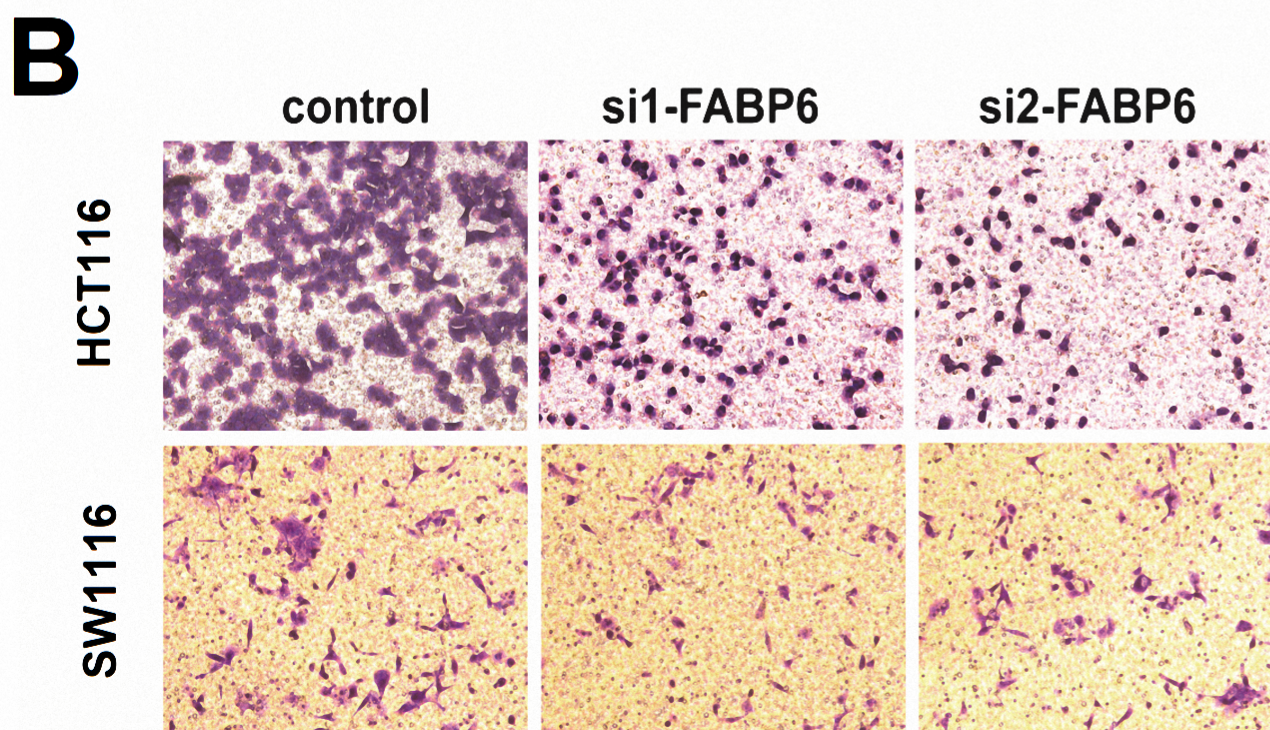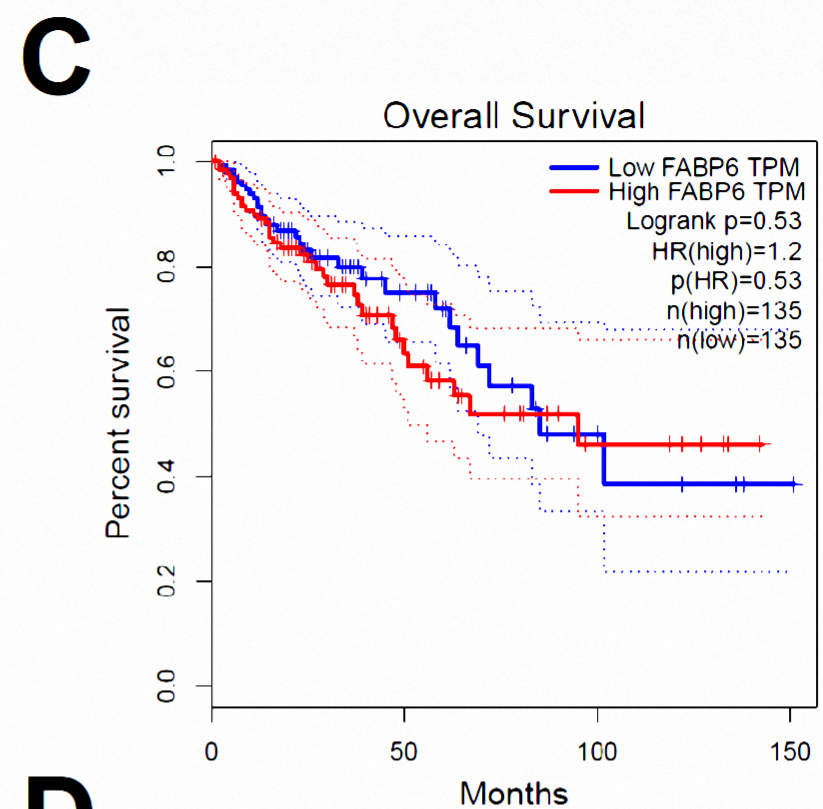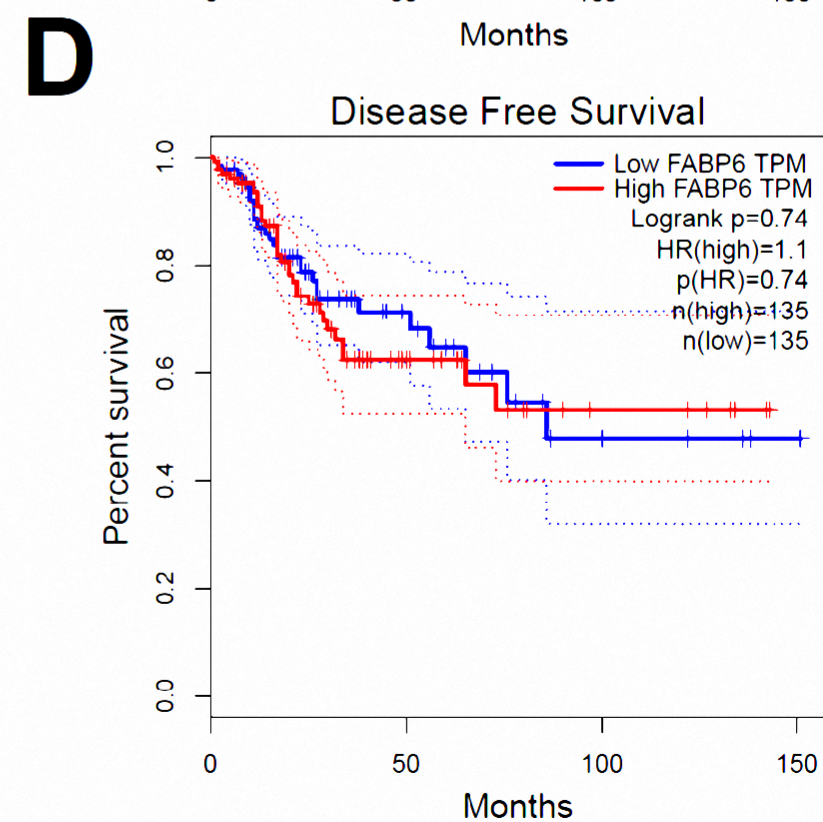

Supplement: Supplementary 9 — Supplementary Figure 9: (A, B) transwell assays showed that FABP6 knockdown could slightly inhibit the migration and invasion abilities of tumor cells (A: migration, B: invasion). (C, D) Survival analysis between FABP6 and OS and DFS of CRC patients. [file 3129765.f9.pdf]

**A**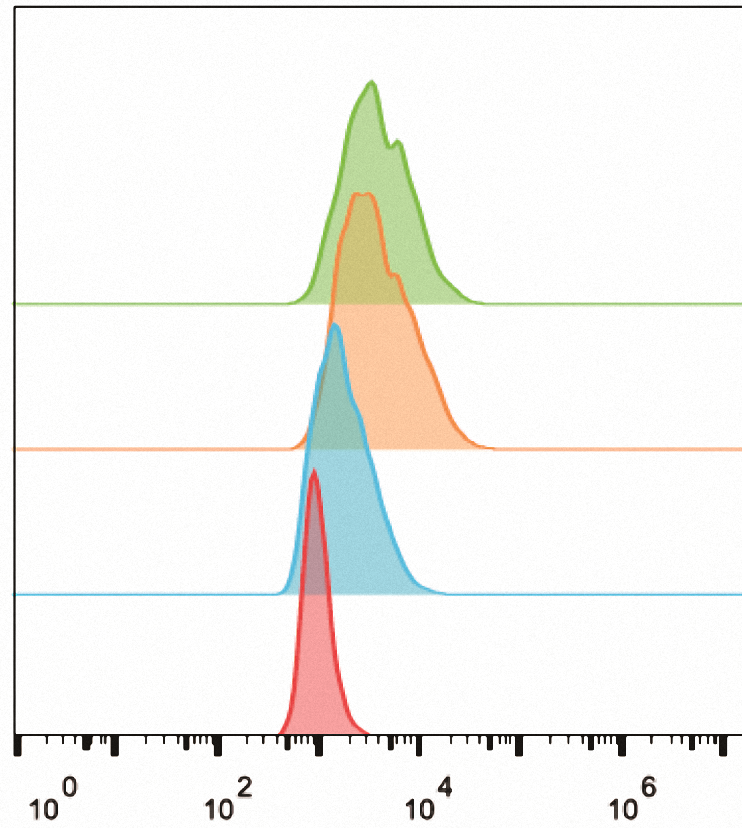**HLA-A/B/C-APC****HCT116****B**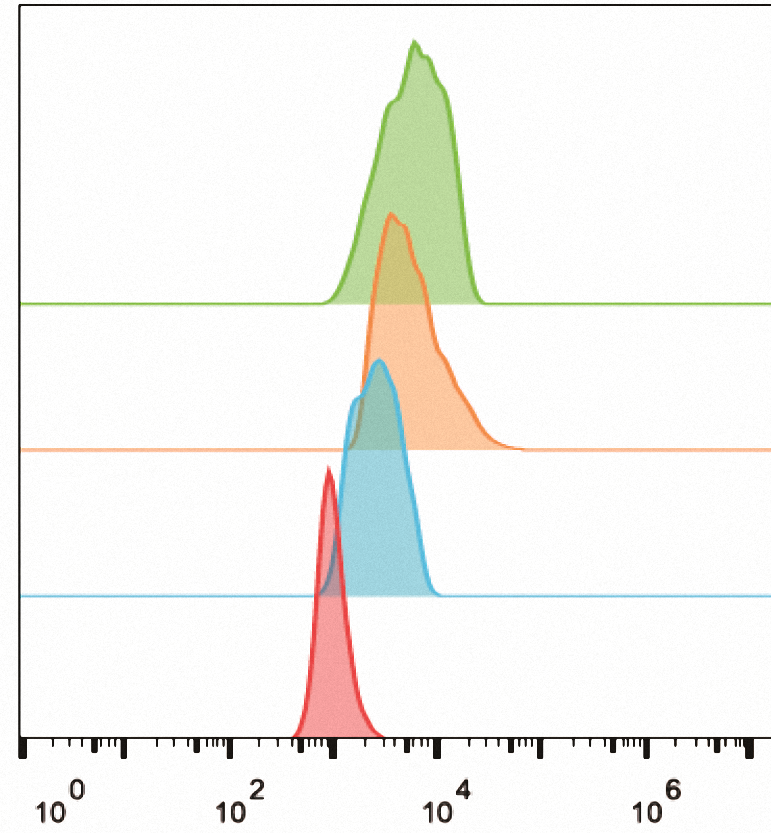**HLA-A/B/C-APC****SW1116**

Supplement: Supplementary 10 — Supplementary Figure 10: mean fluorescence intensity HLA-A/B/C expression with FABP6 knockdown in groups of control, si1-FABP6, and si2-FABP6. [file 3129765.f10.pdf]
