# Supplementary material for: FABP6 Expression Correlates with Immune Infiltration and Immunogenicity in Colorectal Cancer Cells
Source: J Immunol Res. 2022 Aug 17;2022:3129765. doi: 10.1155/2022/3129765 (PMC9403257; doi:10.1155/2022/3129765)
Supplement: Supplementary 11 — Supplementary Table 1: primers used in this study. [file 3129765.f11.pdf]

Supplementary Table 1: Primers used in this study.

Gene	Forward	Reverse
FABP6	ACCGGCAAGTTCGAGATGG	CCTTTTCGATTACATCGCTGGA
CCL4	CTGTGCTGATCCCAGTGAATC	TCAGTTCAGTTCCAGGTCATACA
CCL5	CCAGCAGTCGTCTTTGTCAC	CTCTGGGTTGGCACACACTT
CXCL9	CCAGTAGTGAGAAAGGGTCGC	AGGGCTTGGGGCAAATTGTT
CXCL10	GTGGCATTCAAGGAGTACCTC	TGATGGCCTTCGATTCTGGATT
HLA-A	ACCCTCGTCCTGCTACTCTC	CTGTCTCCTCGTCCCAATACT
HLA-B	CAGTTCGTGAGGTTCGACAG	CAGCCGTACATGCTCTGGA
HLA-C	CCATGAGGTATTTGTGGACCG	TCTCGGACTCTCGTCGTCG
B2M	GAGGCTATCCAGCGTACTCCA	CGGCAGGCATACTCATCTTTT
TAP1	TGCCCCGCATATTCTCCCT	CACCTGCGTTTTCGCTCTTG
TAP2	TGGACGCGGCTTTACTGTG	GCAGCCCTCTTAGCTTTAGCA
